# Bacterial and gut microbiota-derived extracellular vesicles as emerging sources of cancer biomarkers: molecular mechanisms, diagnostic approaches, and therapeutic applications

**DOI:** 10.3389/fcimb.2026.1806945

**Published:** 2026-07-23

**Authors:** Hao Ying

**Affiliations:** 1 Sports Health Technology College, Jilin Sports University, Changchun, Jilin, China; 2 Zhuji People’s Hospital of Zhejiang Province, Zhuji Affiliated Hospital of Shaoxing University, Zhuji, Zhejiang, China

**Keywords:** cancer, diagnostic approaches, gut microbiota, molecular mechanisms, oncology

## Abstract

Extracellular vesicles (EVs) originating from bacteria and gut microbiota have recently been recognized as pivotal agents in the communication between host and microbes, exhibiting considerable promise as innovative biomarkers and therapeutic instruments in the realm of oncology. These nanoscale vesicles encapsulate a heterogeneous array of molecular constituents, including metabolites, proteins, nucleic acids, and toxins, which possess the capacity to influence tumor proliferation, apoptosis, immune responses, and metastasis. Recent investigations underscore their bifunctional nature: specific bacterial EVs can facilitate oncogenesis by altering signaling cascades such as BRCA1/EXO1/TP53BP1 or TGF-β1/Smad, while others may suppress tumor growth through the induction of oxidative stress, mitophagy, or the activation of antitumor immunity via STING or cGAS pathways. The metabolomic and molecular characterizations of bacterial and fecal EVs afford a distinctive, non-invasive perspective into tumor biology and the interactions between host and microbiome. Sophisticated diagnostic methodologies, encompassing targeted metabolomics, high-throughput sequencing, and flow cytometry-based characterization of EVs, have enabled the discovery of EV-derived cancer biomarkers with exceptional specificity and sensitivity. Moreover, engineered EVs that transport therapeutic agents, including prodrugs, microRNAs, or photosensitizers, exhibit significant anticancer efficacy in preclinical experimental models. This review consolidates contemporary understanding regarding the molecular mechanisms, diagnostic capabilities, and therapeutic implications of bacterial and gut microbiota-derived EVs in the context of cancer.

## 1 Introduction

Extracellular vesicles (EVs) are nanoscale structures encased by membranes, secreted by nearly all cellular life forms, and are increasingly acknowledged as essential facilitators of intercellular and inter-organ communication. While extensive research has been conducted on EVs derived from mammalian cells within the context of cancer biology, a growing body of evidence suggests that both bacteria and gut microbiota also release significant quantities of EVs that play an active role in host-microbe interactions. Bacterial extracellular vesicles (BEVs), which encompass outer membrane vesicles from Gram-negative bacteria as well as membrane vesicles from Gram-positive organisms, are known to transport a wide array of bioactive components, including lipopolysaccharides (LPS), peptidoglycans, proteins, metabolites, DNA, and regulatory RNAs. Notably, EVs originating from gut microbiota are not merely incidental products of bacterial metabolism; rather, they constitute biologically active entities that possess the capability to traverse epithelial barriers, infiltrate systemic circulation, and influence remote organs, including neoplastic tissues (Kulp and Kuehn, 2010; Brown et al., 2015; Kaparakis-Liaskos and Ferrero, 2015). Recent investigations have revealed that the molecular architecture of EVs derived from microbiota exhibits significant divergence from that of the overall gut microbiome, indicating that EVs represent a separate functional and taxonomic domain of microbial impact. This differentiation is particularly pertinent within the field of oncology, where nuanced molecular indicators, rather than mere microbial quantity, may be instrumental in the initiation, progression, immune evasion, or therapeutic responsiveness of tumors. Circulating BEVs have been identified in plasma, serum, urine, and various other biofluids, underscoring their stability and potential as viable candidates for biomarkers in liquid biopsy applications (Jeon et al., 2017; Su et al., 2022; Kaisanlahti et al., 2025). In contrast to traditional tumor-derived EVs, microbiota-derived EVs encapsulate data regarding host-microbe interactions and systemic microbial dysbiosis, phenomena that are increasingly recognized as playing a critical role in cancer susceptibility and its associated heterogeneity.

From a mechanistic standpoint, EVs derived from bacteria and gut microbiota exert significant influence on cancer biology via multiple interconnected pathways. These pathways encompass the modulation of both innate and adaptive immune responses, the regulation of inflammatory signaling cascades such as NF-κB, STAT3, and MAPK, the alteration of epithelial barrier integrity, and the metabolic reprogramming that occurs within the tumor microenvironment (TME). Empirical evidence indicates that BEVs can foster pro-tumorigenic inflammation under dysbiotic conditions; conversely, in alternative contexts, they may elicit anti-tumor immune-stimulating effects, thereby highlighting their context-dependent functionalities (Caruana and Walper, 2020; Chronopoulos and Kalluri, 2020; Díaz-Garrido et al., 2021). This duality complicates simplistic interpretations of microbiota–cancer interactions and underscores the necessity for biomarker discovery that is informed by mechanistic understanding. In the realm of precision medicine, there exists a pressing necessity for biomarkers that exhibit minimal invasiveness, dynamic responsiveness, and a mechanistic connection to pathological processes. EVs derived from microbiota meet these stringent requirements by encapsulating microbial activity, host responses, and disease states within a singular nanoscale delivery system. Recent investigations suggest that bacterial DNA, small RNAs (sRNAs), and protein signatures associated with EVs can effectively differentiate cancer patients from healthy individuals with remarkable accuracy, particularly in the context of early-stage malignancies where traditional biomarkers frequently demonstrate limitations (Park et al., 2021; Yang et al., 2022; Tatischeff, 2023). Furthermore, unique signatures of both BEVs and host-derived EVs have been correlated with specific malignancies, including colorectal, gastric, lung, breast, and hepatocellular carcinoma, thereby indicating their potential application in tumor stratification and the assessment of therapeutic efficacy (Yang et al., 2016; Kim et al., 2024; Liang et al., 2024; Gabaran et al., 2026; Yu et al., 2026).

Despite the increasing scholarly interest, the prevailing body of literature remains disjointed. T7o7777here exists a significant and unmet demand for a thorough, cancer-centric synthesis that interconnects molecular mechanisms, diagnostic methodologies, and therapeutic implications of bacterial and gut microbiota-derived EVs. This review is thus meticulously constructed to address this lacuna by rigorously assessing the existing body of evidence concerning microbiota-derived EVs as nascent cancer biomarkers, while underscoring the mechanistic plausibility, translational significance, and clinical applicability. Instead of merely cataloging extant studies, we synthesize findings across diverse experimental paradigms, clinical cohorts, and technological platforms to illuminate consistencies, disputes, and gaps in knowledge. Special emphasis is directed toward methodological hurdles, encompassing EV isolation, assignment of microbial origin, and standardization, which continue to pose significant obstacles to clinical translation. In summary, EVs originating from bacteria and gut microbiota signify a previously overlooked yet profoundly enlightening dimension of biological communication in the context of cancer. Their capability to encapsulate signals derived from microbial and host interactions, persist within the circulatory system, and signify systemic dysbiosis establishes them as formidable candidates for the next generation of cancer biomarkers. Empirical observations within the domain reveal that their paramount utility resides not in supplanting existing biomarkers but in augmenting them, thus improving diagnostic precision, patient stratification, and tailored therapeutic decision-making. Future advancements will hinge on meticulous mechanistic investigations, the establishment of standardized analytical methodologies, and the execution of thoughtfully designed clinical trials to comprehensively actualize the potential of microbiota-derived EVs in precision oncology.

## 2 Biogenesis and characterization of BEVs

### 2.1 Formation mechanisms of BEVs

**Figure 1:**
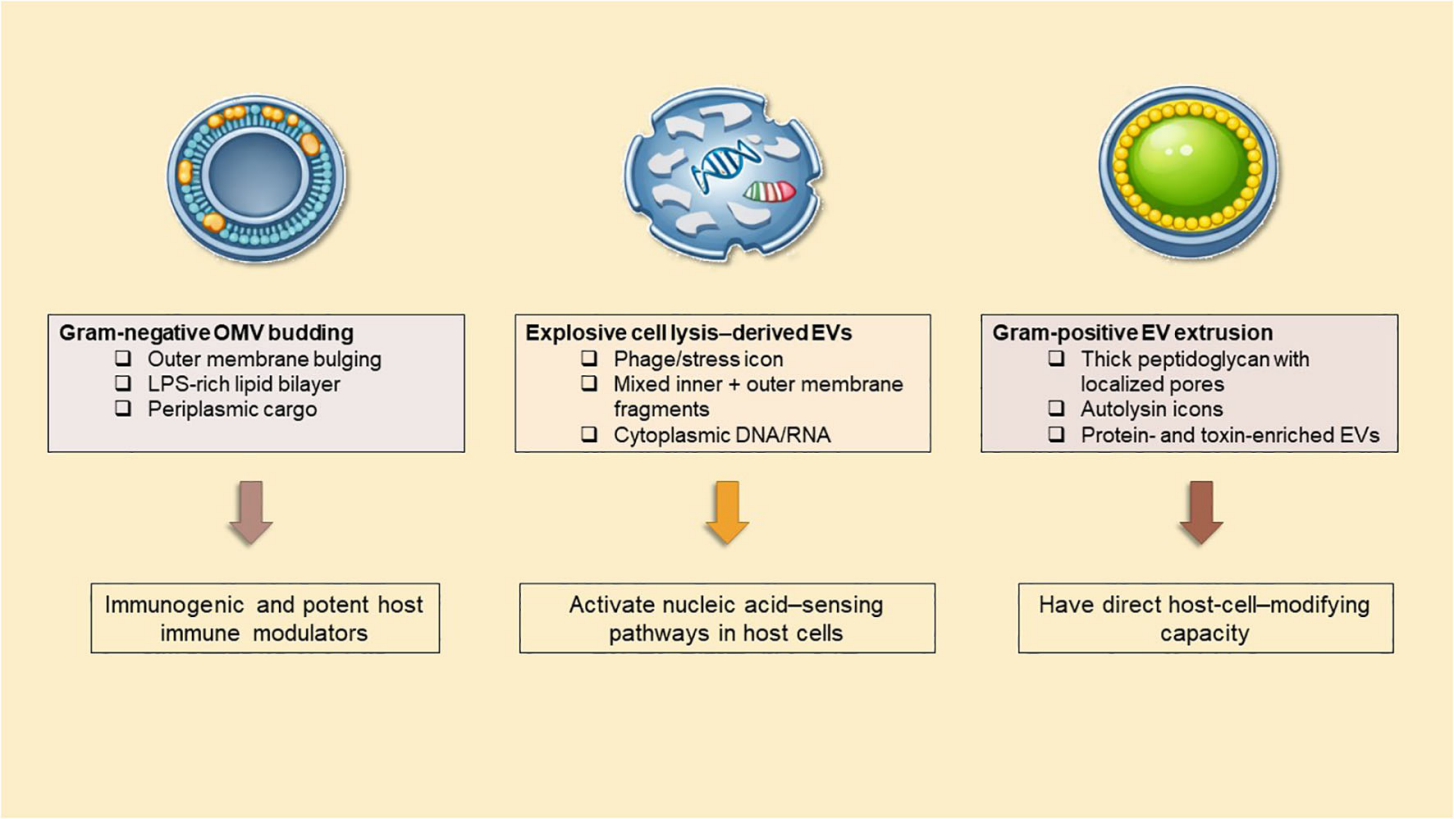
Major biogenesis pathways of bacterial extracellular vesicles (BEVs). This figure illustrates key mechanisms of BEV formation, including outer membrane vesicle (OMV) budding in Gram-negative bacteria, vesicle generation following explosive cell lysis, and vesicle extrusion through the thick cell wall of Gram-positive bacteria. The resulting vesicles exhibit distinct molecular characteristics, including differences in lipid composition, nucleic acid content, protein cargo, and immunogenic properties, which contribute to their diverse roles in host interactions.

BEVs are generated through evolutionarily conserved yet structurally distinct mechanisms that are contingent upon the architectural composition of the bacterial cell envelope and the prevailing environmental conditions. In Gram-negative bacteria, the biogenesis of vesicles predominantly transpires via the development of outer membrane vesicles (OMVs), which emanate from localized outward protrusions of the outer membrane. This phenomenon is mediated by a multitude of interacting factors, including the accumulation of misfolded proteins or peptidoglycan fragments within the periplasmic space, the perturbation of outer membrane–peptidoglycan crosslinking, modifications in LPS composition, and the uneven distribution of phospholipids. These occurrences induce membrane curvature and tension that promote vesicle release while preserving bacterial viability (Kulp and Kuehn, 2010; Schwechheimer and Kuehn, 2015). Beyond the traditional scope of OMVs, bacterial organisms are capable of producing diverse populations of vesicles as a consequence of stress-induced phenomena or phage-mediated explosive cell lysis. In this process, fragments derived from both the inner and outer membranes undergo spontaneous reassembly into vesicular formations that encapsulate cytoplasmic constituents, which include enzymes and nucleic acids. While such vesiculation was initially perceived as a mere consequence of bacterial demise, an increasing body of evidence suggests that explosive vesiculation constitutes a regulated adaptive mechanism in response to environmental stresses, antibiotic exposure, and immune challenges, thereby playing a crucial role in microbial communication and survival (Turnbull et al., 2016; Mozaheb and Mingeot-Leclercq, 2020). In Gram-positive bacteria, the lack of an outer membrane coupled with the existence of a robust peptidoglycan layer was historically assumed to inhibit the release of EVs. Nonetheless, contemporary research indicates that Gram-positive organisms actively excrete EVs via localized enzymatic remodeling of the cell wall, a process facilitated by autolysins and peptidoglycan hydrolases, in conjunction with intracellular turgor pressure. These EVs possess the capability to penetrate the cell wall without inducing cell lysis and frequently encapsulate virulence factors, enzymes, and immunomodulatory molecules, highlighting their biological significance and evolutionary conservation across various bacterial taxa (Lee et al., 2009; Brown et al., 2015) (**Figure 1**).

### 2.2 Physical and molecular characterization of BEVs

BEVs typically exhibit a diameter ranging from 20 to 300 nm, which significantly overlaps with the dimensions of mammalian exosomes and microvesicles, thereby complicating their distinction within intricate biological fluids. Morphologically, BEVs display either spherical or cup-shaped configurations when examined via transmission or cryo-electron microscopy; however, vesicular heterogeneity is prevalent and reflects variations in biogenetic pathways as well as the bacterial source (Schwechheimer and Kuehn, 2015). At the molecular level, BEVs encapsulate a remarkably diverse and selectively enriched array of cargo, which includes LPS, lipoteichoic acids, outer membrane proteins, transporters, enzymes, metabolites, fragments of bacterial DNA, plasmids, and regulatory sRNAs. Proteomic and lipidomic analyses have demonstrated that the loading of cargo into BEVs is not a stochastic process but rather reflects intricate sorting mechanisms that prioritize molecules pertinent to host interaction, immune modulation, and microbial competition. Unlike mammalian EVs, which are commonly characterized using established surface markers such as CD9, CD63, and CD81, there are currently no universally accepted markers for bEV identification. Most studies therefore rely on bacterial species-specific components, including LPS, peptidoglycan, and outer membrane proteins such as OmpA and OmpC, for vesicle characterization. In addition, bacterial nucleic acid signatures such as 16S rRNA and bacterial DNA fragments are increasingly used to support bEV identification. However, the absence of standardized markers and characterization protocols remains a significant methodological limitation that may affect reproducibility and cross-study comparability (Jeon et al., 2017; Díaz-Garrido et al., 2021). Importantly, the lipid composition of BEVs frequently exhibits significant discrepancies from that of the parental bacterial membrane, characterized by an enrichment of phosphatidylethanolamine, cardiolipin, or modified lipid A species, which may have implications for vesicle stability, cellular uptake, and immunogenicity. Despite these advancements, standardized molecular indicators for BEVs remain inadequately characterized, particularly when juxtaposed with mammalian EVs, thereby presenting significant obstacles for reproducibility and interpretation across various studies. This constraint is particularly pertinent in the realm of cancer biomarker research, in which accurate attribution of EV origin and cargo is indispensable for successful clinical translation (Kuehn and Kesty, 2005; Zhang et al., 2024).

### 2.3 Methods for isolation of BEVs

The isolation of BEVs constitutes a pivotal factor influencing analytical precision and continues to represent a significant technical obstacle within this domain. Differential ultracentrifugation is the predominant methodology utilized, facilitating the separation of EVs predicated on their size and density via a series of sequential centrifugation phases. Although this technique is broadly accessible, it is characterized by a high labor demand and is vulnerable to the simultaneous isolation of protein aggregates, lipoproteins, and vesicles derived from the host, particularly when employed with intricate clinical specimens (Li et al., 2017). Size-exclusion chromatography has emerged as a supplementary or alternative methodology that enhances vesicle purity and maintains the structural integrity of EVs by segregating particles according to their hydrodynamic radius. Nevertheless, this technique frequently results in diminished vesicle recovery and may prove less advantageous for extensive clinical investigations. Density gradient ultracentrifugation offers enhanced specificity by leveraging variations in buoyant density; however, it is technically intricate and time-consuming, which restricts its routine implementation in translational research.

Commercially available precipitation-based isolation kits provide both convenience and scalability, leading to their increasing application in clinical and high-throughput environments. However, these techniques often result in the co-precipitation of non-vesicular contaminants and are inadequately suited for mechanistic investigations or biomarker identification that necessitate high levels of purity. As a result, the selection of an isolation methodology must be meticulously aligned with the specific objectives of the study, and methodological transparency is imperative for ensuring reproducibility and facilitating cross-study comparisons (Ramirez et al., 2018).

The complexity of biological matrices significantly affects the isolation, recovery, and characterization of BEVs. Various sample types, such as plasma, serum, stool, urine, and tissue-associated microbiota, exhibit considerable differences in terms of protein content, host-derived EV presence, microbial load, viscosity, and the presence of contaminants. For instance, plasma and serum have high levels of circulating proteins, lipoproteins, and mammalian EVs that complicate the purification of BEVs. In contrast, stool samples are rich in microbial populations, dietary residues, and other particulate matter, which challenge the enrichment and purity of vesicles. Urine samples may yield lower vesicle concentrations and exhibit inconsistent physicochemical properties, while tissue-associated samples often require extra processing to eliminate cellular debris and components of the extracellular matrix.

As a result, the efficacy of isolation techniques, such as ultracentrifugation, density gradient separation, filtration, and size-exclusion chromatography, varies greatly with the sample type, and no single method currently achieves optimal recovery and specificity for all biological matrices. To enhance vesicle purity and reduce contamination from host EVs, protein aggregates, and microbial debris, it is often necessary to employ combined or sequential isolation methods.

Despite the promising therapeutic potential of BEVs, important safety concerns remain insufficiently addressed. Because BEVs naturally contain immunogenic bacterial components such as LPS, peptidoglycan, bacterial toxins, and outer membrane proteins, they may trigger excessive inflammatory responses, systemic toxicity, or unintended immune activation following administration. In addition, the long-term biodistribution, immunogenicity, and off-target effects of therapeutic BEVs remain poorly understood, particularly in complex *in vivo* systems. Several recent studies have attempted to improve biosafety through genetic engineering, endotoxin reduction, membrane modification, or the development of synthetic and hybrid vesicle systems designed to preserve therapeutic efficacy while minimizing toxicity. Nevertheless, standardized safety evaluation protocols and comprehensive preclinical biosafety studies are still lacking, representing a major challenge for the future clinical translation of BEV-based therapies.

### 2.4 Evidence from preclinical and clinical studies

Recent investigations have elucidated the presence of BEVs and microbial-derived small RNAs (msRNAs) within human plasma, establishing them as a novel and mechanistically significant category of circulating cancer biomarkers.

Multi-cohort studies encompassing a cohort of over 1,100 individuals reveal markedly elevated levels of BEVs in cancer patients in comparison to control subjects, in conjunction with a quantifiable contribution from microbial RNAs to the circulating RNA milieu (Mehmood and Danaf, 2026). Notably, specific BEV–msRNA signatures exhibit superior diagnostic accuracy for the detection of early-stage cancers, surpassing the performance of conventional tumor markers such as CEA and CA19-9. Functional analyses indicate that msRNAs associated with BEVs actively influence tumor biology by activating NF-κB and STAT3 signaling pathways while concurrently inhibiting tumor suppressor mechanisms, thereby fostering inflammatory and oncogenic responses. The remarkable stability of BEVs within the circulatory system further substantiates their potential clinical utility as reliable liquid biopsy instruments for cancer detection and precision monitoring (Mehmood and Danaf, 2026) (**Table 1**). Growing empirical evidence suggests that components derived from bacteria are present in human circulation and contribute to microbial signatures that are specific to various diseases (Fujikawa et al., 2025). The early identification of gastric cancer poses significant challenges, primarily due to the absence of dependable non-invasive biomarkers. Another study explored the potential of bacteria-derived EVs as innovative metagenomic biomarker sources for gastric cancer by utilizing stool, urine, and serum samples derived from a large cohort of both patients and controls. Metagenomic profiling of EV-associated bacterial DNA unveiled noteworthy discrepancies in microbial diversity and composition between individuals diagnosed with gastric cancer and healthy subjects across all sample types. Genus-level microbial signatures were employed to develop diagnostic prediction models, among which the urine-based EV model exhibited the most robust diagnostic performance in comparison to stool- and serum-based methodologies. These findings shown the diagnostic superiority of urine-derived BEVs and advocate for their utilization as non-invasive liquid biopsy instruments. Collectively, the study emphasizes the potential of bacteria-derived EVs, in conjunction with metagenomic analysis, for the early detection of gastric cancer and subsequent clinical translation (Park et al., 2021). BEVs have emerged as significant nanoscale mediators that facilitate communication between the host and the gut microbiome, providing functional insights that extend beyond traditional microbiome profiling methodologies. A research sought to address a pivotal lacuna by exploring the role of BEVs derived from the gut microbiome in individuals diagnosed with solid tumors. Through comparative proteomic and metagenomic evaluations of fecal BEVs obtained from cancer patients juxtaposed with those from healthy controls, a diminished microbial richness and diversity was observed in the patient cohort, accompanied by a seemingly paradoxical enrichment of functionally diverse BEVs proteomes linked to metabolic and redox-related pathways. Machine learning-based classification techniques exhibited enhanced accuracy when employing BEV-associated signatures as opposed to relying solely on fecal microbiome data, thereby emphasizing the supplementary diagnostic potential of BEVs. In summary, these findings indicated the notion of BEVs as distinct functional entities and accentuate the necessity of integrating BEV analyses with conventional microbiome approaches to facilitate advancements in cancer biomarker discovery and functional oncology research (Mishra et al., 2025b).

**Table 1:** Overview of bacterial and microbiota-derived extracellular vesicles and tumor exosomes as diagnostic and therapeutic biomarkers in cancer.

Biomarker type	Sample type	Cohort	Findings	Diagnostic/prognostic value	Ref
Bacterial extracellular vesicles (BEVs) and microbial small RNAs (msRNAs)	Plasma	>1,100 patients with various cancers	BEV levels 2.8–3.5× higher in cancer cases; msRNAs comprise up to 1.6% of total plasma RNA	BEV–msRNA panels detected early-stage cancer with AUC 0.87–0.94, outperforming CEA and CA19-9; stable in circulation >72 h	(Mehmood and Danaf, 2026)
Bacterial DNA	Serum EVs	89 gastric cancer patients; 25 healthy donors	Reduced Bacteroidetes and *Actinobacteria*, increased Firmicutes in GC patients	BAF index (Bacteroidetes + Actinobacteria/Firmicutes) showed high sensitivity for GC detection; associated with advanced tumor stage and poor prognosis	(Fujikawa et al., 2025)
Bacterial DNA	Serum EVs	88 RCC patients; 10 healthy donors	Three dominant bacteria: *Bacteroidia*, TM7-1, Sphingomonadales; BTS index developed	BTS index showed high sensitivity in discovery and validation cohorts; high Bacteroidia levels linked to worse progression-free and overall survival under nivolumab	(Uemura et al., 2023)
Bacterial extracellular vesicles	Urine, stool, serum	453 subjects (GC 181; controls 272)	Significant differences in microbial diversity and composition between GC patients and controls; genus-level variations identified	Urine-based EV metagenomic model had the highest diagnostic performance among all sample types	(Park et al., 2021)
Bacterial extracellular vesicles (proteins, mRNAs, metabolites, lipids)	Feces	Solid tumor patients and healthy controls	Decreased microbiota richness and diversity in patients; BEV proteomes more diverse and enriched in metabolic, nucleotide-binding, and oxidoreductase proteins	Machine learning classification based on BEVs achieved 100% accuracy vs. 93% with fecal microbiome alone	(Mishra et al., 2025b)
Commensal bacteria-derived EVs	*In vitro* DCs; mouse tumor models	Solid tumor mouse models	*Lactobacillus* EVs induced IL-12 in DCs, promoted Th1/Tc1 differentiation, and reduced tumor growth; enhanced effect of anti-PD-1 and oxaliplatin	Potential immunotherapy: EVs suppressed tumor growth, dependent on IL-12 and TLR9 signaling	(Kim and Bae, 2023)
Tumor cell-derived exosomes (TDEs)	Serum	Mouse tumor models; patient serum validation	Nucleolin-positive exosomes detected using aptamer-functionalized Au@Pt nanozyme-coated bacteria; colorimetric aptasensor enabled rapid, sensitive detection	Distinguished tumor patients from healthy and inflammatory cases with 100% accuracy; LOD 40 particles/mL; monitored PTT and chemotherapy response	(Huang et al., 2025)

BEVs, bacterial extracellular vesicles; msRNAs, microbial-derived small RNAs; EVs, extracellular vesicles; GC, gastric cancer; RCC, renal cell carcinoma; BAF, Bacteroidetes + Actinobacteria/Firmicutes index; BTS, Bacteroidia, TM7-1, and Sphingomonadales index; TDEs, tumor cell-derived exosomes; PTT, photothermal therapy; DCs, dendritic cells; LOD, limit of detection; AUC, area under the curve; IFN-γ, interferon-gamma; Th1/Tc1, T-helper 1/cytotoxic T cells.

Commensal microbiota-derived EVs have been recognized as significant modulators of antitumor immunity. A study elucidated a particular Lactobacillus strain and its corresponding EVs as potent inducers of interleukin-12 synthesis in dendritic cells, while notably avoiding the concurrent induction of the immunosuppressive cytokine IL-10 (Kim and Bae, 2023). Dendritic cells stimulated by *Lactobacillus* EVs facilitated the differentiation of Th1 and Tc1 cells, which culminated in augmented antitumor immune responses. In murine models of tumors, both oral and systemic administration of these EVs markedly inhibited tumor growth by promoting IFN-γ synthesis and enhancing the cytotoxic effector functions of tumor-infiltrating T cells. Importantly, Lactobacillus EVs exhibited a synergistic effect when combined with anti-PD-1 immunotherapy and oxaliplatin. From a mechanistic standpoint, bacterial DNA encapsulated within EVs was found to activate TLR9 signaling pathways, and the blockade of IL-12 or the deficiency of TLR9 completely negated the observed antitumor effects (Kim and Bae, 2023). Tumor cell-derived exosomes (TDEs) represent a novel class of liquid biopsy biomarkers that exhibit significant potential for the early diagnosis of cancer and the monitoring of therapeutic interventions; however, their clinical applicability is constrained by their low prevalence and the absence of universally applicable markers. This investigation sought to overcome these limitations by specifically targeting nucleolin-positive exosomes through the implementation of a colorimetric aptasensor that utilizes nucleolin aptamer-functionalized Au@Pt nanozyme-coated bacteria (apAS-E.coli-Au@Pt) as an amplification mechanism for signal detection. By capitalizing on the extensive surface area and catalytic properties of the *in situ* synthesized nanozyme, the proposed system accomplished ultra-sensitive detection of exosomes, achieving a detection threshold of 40 particles/mL. Utilization in murine models facilitated precise monitoring of tumor progression as well as responses to photothermal therapy and chemotherapy. Notably, the aptasensor was able to accurately differentiate cancer patients at varying stages from healthy individuals and those with inflammatory conditions, achieving a remarkable accuracy rate of 100%, thereby underscoring its potential as a non-invasive modality for early cancer detection and treatment assessment (Huang et al., 2025).

In addition to BEVs, increasing attention has also been directed toward host- and tumor-derived extracellular vesicles, which similarly contribute to intercellular communication and cancer progression within the tumor microenvironment.

Through the application of 16S rRNA metagenomic analysis on serum EVs collected from individuals diagnosed with gastric cancer and from healthy control subjects, this investigation identified a distinctive bacterial DNA profile characterized by a decrease in Bacteroidetes and Actinobacteria coupled with an increase in Firmicutes among cancer patients (Fujikawa et al., 2025). In light of these observed alterations, a composite BAF index was formulated, exhibiting high sensitivity for the detection of gastric cancer across both discovery and validation cohorts. Notably, elevated values of the BAF index were correlated with advanced stages of tumors, unfavorable prognostic outcomes, and an immunosuppressive TME, indicating biological significance that transcends mere diagnostic efficacy. Collectively, these findings indicated the notion that signatures of circulating microbiota-derived EVs serve as clinically relevant biomarkers for the purposes of screening, prognostication, and characterization of tumor immunity in gastric cancer (Fujikawa et al., 2025).

An investigation assessed the clinical relevance of circulating bacterial DNA in individuals diagnosed with renal cell carcinoma by employing 16S rRNA metagenomic profiling of serum EVs. Examination of specimens from RCC patients in conjunction with healthy donors revealed three predominant bacterial taxa, Bacteroidia, TM7-1, and Sphingomonadales, that effectively differentiated cancer patients from control subjects. By integrating these bacterial signatures, a composite BTS index was devised, which exhibited elevated diagnostic sensitivity across both discovery and validation cohorts, thereby underscoring its potential utility as a screening biomarker. Importantly, increased concentrations of Bacteroidia-derived DNA in serum EVs were significantly correlated with diminished progression-free and overall survival rates in patients undergoing nivolumab therapy, thereby establishing a link between circulating BEVs signatures and therapeutic outcomes. Collectively, these results underscore the potential of circulating bacteria-derived DNA as a valuable diagnostic and prognostic biomarker in the context of renal cell carcinoma (Uemura et al., 2023).

## 3 Molecular cargo of BEVs in cancer

**Figure 2:**
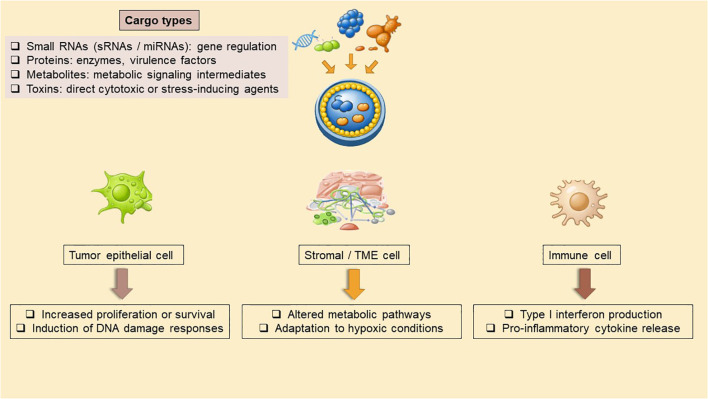
Molecular cargo and functional effects of bacterial extracellular vesicles within the tumor microenvironment. This figure illustrates the delivery of diverse bacterial extracellular vesicle (BEV) cargos, including small RNAs, proteins, metabolites, and toxins, to tumor epithelial, immune, and stromal cells. Following cellular uptake, BEVs activate multiple signaling and sensing pathways that contribute to context-dependent effects such as modulation of cell proliferation, immune polarization, metabolic reprogramming, and remodeling of the tumor microenvironment. Bidirectional arrows indicate that these effects may promote or suppress tumor progression depending on the microbial source and host conditions.

EVs derived from bacteria encompass a heterogeneous and intricate assortment of molecular constituents, including proteins, metabolites, and nucleic acids, which collectively influence processes associated with cancer by altering host cellular signaling pathways and acting as prospective biomarkers (**Figure 2**).

### 3.1 Proteins, metabolites, and nucleic acids (miRNA, mRNA, toxins)

BEVs encapsulate proteins that are representative of their progenitor microorganisms, encompassing enzymes, adhesins, and virulence factors capable of perturbing host cellular biology. Proteomic investigations have elucidated that these vesicular proteins participate in immune modulation, inflammatory responses, and carcinogenic mechanisms (Kaparakis-Liaskos and Ferrero, 2015; Dhital et al., 2021). Additionally, metabolites contained within BEVs include short-chain fatty acids (SCFAs), polyamines, and lipids that significantly influence host metabolic pathways and signaling networks pertinent to tumorigenesis (Berleman and Auer, 2013; Gao and van der Veen, 2020). Furthermore, BEVs are known to transport nucleic acids, such as sRNAs, mRNA fragments, and occasionally sequences derived from plasmids. These bacterial RNAs, which include miRNA-like entities, can be transferred to host cells to modulate gene expression at the post-transcriptional level, thereby potentially impacting cellular proliferation, apoptosis, and immune responses (Koeppen et al., 2016; Tsatsaronis et al., 2018). Certain EVs also carry bacterial toxins such as cytolethal distending toxin (CDT) and VacA from *Helicobacter pylori*, that directly compromise cellular integrity and facilitate mutagenesis (Olofsson et al., 2010).

### 3.2 Metabolomic signatures as emerging sources of cancer biomarkers

Metabolomic profiling of BEVs has revealed unique signatures that are significantly associated with the presence and progression of cancer. Alterations in lipidomic profiles, including increased levels of LPS components and distinctive fatty acid species, have been linked to colorectal and pancreatic malignancies (Yang et al., 2019). SCFAs such as butyrate and propionate, when aberrantly represented in vesicular cargo, serve as indicators of dysbiosis and modified microbial metabolism within the TME and have been proposed as noninvasive biomarkers for early detection (Louis and Flint, 2017). Additionally, BEVs contain metabolites that are involved in oxidative stress and nucleotide metabolism, which are critical hallmarks of cancer cells, thereby presenting an emerging array of diagnostic candidates (Bernhardt et al., 2025).

### 3.3 Overview of molecular cargo of BEVs in cancer

The early identification of gastric carcinoma is of paramount importance; nevertheless, the underlying mechanisms of its pathogenesis remain inadequately elucidated. This investigation examined the potential of microbial-derived EVs as biomarkers throughout various phases of gastric carcinogenesis. Microbial EVs were extracted from gastric juice, saliva, serum, and urine samples of 141 participants, comprising individuals diagnosed with gastric cancer or dysplasia as well as healthy controls, and subsequently analyzed utilizing next-generation sequencing methodologies. The findings indicated substantial modifications in both alpha and beta diversity of microbial EVs across the progression of the disease. Distinctive microbial signatures were discerned in all sample types, with particular species, *Cutibacterium acnes, Streptococcus oralis, Pseudomonas antarctica, Ralstonia insidiosa, and Pseudomonas yamanorum*, exhibiting abundance patterns that are specific to disease stages. These results indicated that microbial EVs derived from liquid biopsy specimens may function as non-invasive biomarkers for the early detection and ongoing assessment of gastric cancer progression (You et al., 2025) (**Table 2**).

**Table 2:** Overview of molecular cargo of bacterial EVs in cancer.

Study/topic	Sample/model	Key methods	Findings	Ref
Distinct microbial signatures of liquid biopsy samples during gastric carcinogenesis and EV analysis	141 participants (132 gastric cancer/dysplasia, 9 healthy controls); gastric juice, saliva, serum, urine	EV isolation, NGS of bacterial DNA, alpha & beta diversity analysis	- Alpha diversity increased in gastric juice & serum of disease groups - Beta diversity differed among patient groups - Specific bacterial species (Cutibacterium acnes, Streptococcus oralis, Pseudomonas antarctica, Ralstonia insidiosa, Pseudomonas yamanorum) showed stage-specific abundance patterns	(You et al., 2025)
Fusobacterium nucleatum-derived OMVs promote immunotherapy resistance via tryptophan metabolism in TAMs	HNSCC models; *in vivo* mouse studies; patient data	*F.nucleatum* OMV isolation, TAM functional assays, TDO2/AHR pathway analysis, anti-PD-1 treatment	- F.n-OMVs induced immunosuppressive TAM phenotypes - Decreased cytotoxic T cell infiltration - TDO2/AHR pathway activated by tryptophanase in OMVs - TDO2 inhibitor improved anti-PD-1 therapy	(Li et al., 2025)
Proteomic profiling of EVs reveals potential biomarkers for H. pylori infection and gastric cancer	AGS gastric epithelial cells infected with high- and low-virulence H. pylori (strains TN2wt and Tx30a)	EV isolation (ultracentrifugation), LC-MS/MS proteomic analysis, bioinformatics	- 107 and 55 differentially expressed proteins in EVs from TN2wt and Tx30a-infected cells vs. non-infected - HpEVs contain proteins essential for H. pylori survival and virulence - TN2wt-infected AGS EVs enriched with GC-related proteins (ATP6V0A1, GAPDH, HINT1, LYZ, RBX1)	(Subsomwong et al., 2025)
Proteomic landscape of stool-derived EVs in patients with pre-cancerous lesions and CRC	Stool samples from healthy volunteers and CRC patients	EV isolation (SEC + ultrafiltration), alternative concentration methods, mass spectrometry proteomics	- No differences in recovery or contamination among concentration methods - Human-derived proteins more abundant than bacterial in EVs - Pancreatic enzymes abundant; zymogen granules likely co-isolated - Identified 10 surface proteins as candidates for colon-derived EV purification	(Northrop-Albrecht et al., 2025)
Analysis of gut microbiome using urinary EVs in CRC patients	Urine samples from 91 CRC patients and 116 healthy controls	EV isolation, DNA extraction, 16S rRNA NGS	- CRC patients had lower alpha-diversity - Beta-diversity differed significantly between CRC and controls - Specific microbiome signatures associated with CRC - No significant differences based on cancer stage or tumor location	(Yoon et al., 2023)
Extracellular vesicles from CRC cells promote metastasis via NOD1 signaling	CRC cell-derived EVs, murine liver metastasis model, plasma EVs from CRC-LM patients	EV isolation, macrophage activation assays, murine CRC-LM model, NOD1-deficient mice, patient plasma EVs analysis	- CRC-EVs activate NOD1 in macrophages - NOD1-activated macrophages secrete inflammatory cytokines/chemokines, promoting CRC cell migration - CDC42 delivered by CRC-EVs activates NOD1 in macrophages - High NOD1 expression correlates with poor CRC-LM prognosis	(Wei et al., 2022)
Bacterial EVs affect endocrine therapy in MCF7 cells	MCF7 breast cancer cells; microbiome samples from breast cancer patients and healthy controls	Microbiome sequencing, incubation of MCF7 cells with tamoxifen ± *Klebsiella* EVs, qRT-PCR, Western blot	- Klebsiella abundant in breast cancer, especially luminal A subtype - K. pneumoniae EVs enhance tamoxifen efficacy - Mechanism involves Cyclin E2 and p-ERK	(An et al., 2021)
Inhibitory impacts of Lactobacillus rhamnosus GG-derived EVs on hepatic cancer cells	HepG2 liver cancer cells	EV isolation (ultrafiltration), TEM, MTT assay, semi-quantitative RT-PCR for bax/bcl-2	- LDEVs at 100 μg/ml show significant cytotoxicity - Increased bax/bcl-2 ratio after 50–100 μg/ml LDEVs - Induces apoptosis in HepG2 cells	(Behzadi et al., 2017)
Engineered lung cell-targeting and SLC7A11 siRNA-expressing BEVs impair NSCLC progression	NSCLC cell lines (NCI-H2122, NCI-H647), xenograft mouse models	TCGA/GEO data analysis, qPCR, Western blot, immunofluorescence, TEM/NTA of BEVs, *in vivo* toxicity assays	- SLC7A11 upregulated in NSCLC - Knockdown impairs proliferation, migration, invasion - BEVs engineered with lung-targeting peptide and siSLC7A11 efficiently deliver siRNA - Induces ferroptosis and inhibits tumor growth/metastasis	(Wan et al., 2025)
Engineered bacterial OMVs enhance tumor immunotherapy via TME remodeling and CD73 siRNA delivery	Tumor-bearing mice; engineered OMVs	Genetic/chemical engineering of OMVs, HAase modification, PBA surface targeting, siCD73 delivery, *in vivo* efficacy assays	- OMVs degrade HA-rich ECM and enhance tumor penetration - CD73 silencing suppresses tumor and CAF migration/invasion - ECM degradation alleviates hypoxia and inhibits angiogenesis - OMVs stimulate M1 macrophage polarization, DC maturation, NK and cytotoxic T cell infiltration	(Cheng et al., 2026)
Metabolome of fecal EVs in patients with malignant solid tumors	Fecal samples from 28 cancer patients and 7 healthy controls	EV isolation, LC-MS-based targeted metabolomics, Metabolite Set Enrichment Analysis, ROC analysis	- L-glutamic acid enriched in cancer patients; guanine and N-acetylneuraminate depleted - Altered pathways: arginine biosynthesis, glyoxylate/dicarboxylate metabolism, branched-chain amino acids, unsaturated fatty acids - Distinct metabolic phenotypes identified between patients and controls	(Mishra et al., 2025a)
Tetranuclear polypyridylruthenium(II) complexes as selective stains for monocytic and epithelial lung carcinoma EVs	THP-1 monocytic cells, A549 lung carcinoma cells	Flow cytometry, EV staining with Rubb7-TNL and Rubb7-TL phosphorescent dyes, TEM	- LPS-stimulated THP-1 cells produced larger/more EVs - Stains selectively differentiate EVs from liposomes/cell debris - Two EV subpopulations distinguished by biochemical differences - A549 EV staining patterns similar to THP-1 EVs	(Wardhani et al., 2024)
Bacterial EVs induce oxidative stress and mitophagy in HT-29 colon cancer cells	HT-29 colon cancer cell line	BEVs from *E. coli* A5922, ROS assays, PINK1 expression, mitophagy assessment, Akt/mTOR pathway analysis	- BEVs trigger oxidative stress and mitophagy - Cytotoxicity and growth inhibition observed in HT-29 cells - Mitophagy linked to PINK1 upregulation and mTOR pathway activation	(Marzoog et al., 2023)
Engineered *E. coli* OMVs with overexpressed pre-miRNA as cancer therapy nanocarriers	Breast cancer models (*in vitro* and *in vivo*)	OMV engineering with tRNALys-pre-miRNA, cell uptake assays, target gene expression analysis, *in vivo* tumor growth studies	- OMVs inherit overexpressed pre-miRNA from bacteria - Delivered pre-miRNA processed into mature miRNA in host cells - Target oncogene CXCR4 downregulated - Tumor proliferation significantly inhibited	(Cui et al., 2022)
EV-mediated IVT mRNA delivery for HER2+ breast cancer using prodrug CB1954	HER2+ breast cancer xenografts in athymic mice	EV loading with IVT HChrR6 mRNA, prodrug tretazicar (CB1954), *in vivo* tumor growth assessment, fluorescence detection	- IVT mRNA-loaded EVs delivered HChrR6 specifically to tumors - CB1954 prodrug activated in tumor cells causing growth arrest - No off-target toxicity observed	(Forterre et al., 2020)

RC, colorectal cancer; HCC, hepatocellular carcinoma**;** OSCC, oral squamous cell carcinoma; EVs, extracellular vesicles; OMVs, outer membrane vesicles; SyBV, synthetic bacterial vesicles; BEVs, bacterial extracellular vesicles; TAMs, tumor-associated macrophages; NSCLC, non-small cell lung cancer; siRNA, small interfering RNA; HA, hyaluronic acid; ECM, extracellular matrix; TME, tumor microenvironment; IVT, *in vitro* transcribed; GDEPT, gene-delivered enzyme prodrug therapy; ROC, receiver operating characteristic; NOD1, nucleotide-binding oligomerization domain 1; AHR, aryl hydrocarbon receptor; TDO2, tryptophan-2,3-dioxygenase 2; TEM, transmission electron microscopy.

Response to immunotherapy in head and neck squamous cell carcinoma (HNSCC) exhibits significant limitations, with the oral bacterium *Fusobacterium nucleatum* being implicated in the mechanisms of tumor immune evasion. An investigation elucidated that OMVs sourced from *F. nucleatum* (F.n-OMVs) facilitate HNSCC advancement through the induction of immunosuppressive tumor-associated macrophages (TAMs), consequently resulting in diminished cytotoxic T-cell infiltration *in vivo*. Mechanistically, TAMs assimilated bacterial tryptophanase from F.n-OMVs, thereby activating the tryptophan-2,3-dioxygenase 2/aryl hydrocarbon receptor (TDO2/AHR) signaling pathway, which subsequently upregulated immunosuppressive cytokines and immune checkpoint molecules. The inhibition of TDO2 significantly augmented the efficacy of anti-PD-1 therapies in tumor-bearing murine models (Li et al., 2025). Clinically, the expression levels of TDO2, along with the abundance of F. nucleatum, were predictive of immunotherapy outcomes in patients diagnosed with HNSCC. These findings unveil a microbiota-mediated mechanism underlying immunotherapy resistance, accentuating the pivotal role of bacterial OMVs in the modulation of tumor immunity and presenting prospective targets for enhancing therapeutic efficacy (Li et al., 2025). *Helicobacter pylori* infection constitutes a significant risk factor for the development of gastric cancer, particularly within the region of Eastern Asia.

Another study evaluated EVs derived from the gut microbiome present in urine as prospective non-invasive biomarkers for the detection of CRC. Urinary EVs were extracted from a cohort of 91 CRC patients and 116 healthy controls, followed by the examination of bacterial DNA utilizing 16S rRNA next-generation sequencing methodologies. The CRC patient cohort demonstrated unique microbial signatures characterized by a modified abundance of specific taxa in comparison to the control group. Alpha-diversity measures were significantly diminished in CRC patients, whereas beta-diversity exhibited notable differences between the groups, thereby indicating overarching shifts within the microbial community. Variations in microbial diversity were not uniformly correlated with the stage of CRC or the anatomical location of the tumor. These outcome demonstrated that gut microbiome-derived EVs in urine may reflect disease-specific microbial modifications and hold potential as non-invasive biomarkers for CRC diagnosis, thereby presenting a promising liquid biopsy strategy for early disease detection and monitoring (Yoon et al., 2023).

The gut and tumor-associated microbiome possess the capacity to affect the progression of breast cancer as well as the response to therapeutic interventions. A research team examined the influence of EVs derived from *Klebsiella pneumoniae* on endocrine therapy in MCF7 breast cancer cell lines. Microbiome analysis demonstrated that the abundance of Klebsiella was significantly elevated in individuals diagnosed with breast cancer, particularly among those exhibiting the luminal A subtype, in comparison to healthy control subjects. Functional assays indicated that EVs sourced from *K. pneumoniae* augmented the anti-hormonal efficacy of tamoxifen in MCF7 cells. Mechanistically, this amplification of therapeutic efficacy was facilitated through the modulation of Cyclin E2 and phosphorylated ERK (p-ERK), thereby implicating the regulation of cell cycle and survival signaling pathways. These findings shown the functional significance of microbiome-derived EVs in the context of breast cancer treatment and propose that Klebsiella-derived EVs may constitute a novel determinant influencing the response to endocrine therapy, thereby providing valuable insights for the development of therapeutic strategies (An et al., 2021). BEVs) are increasingly recognized as pivotal mediators of host–pathogen interactions as well as potential agents in anticancer therapies. Another study aimed to elucidate the impact of EVs isolated from *Lactobacillus rhamnosus* GG (LDEVs) on the apoptotic processes occurring in HepG2 liver cancer cells. LDEVs were extracted utilizing ultrafiltration techniques and subsequently validated through transmission electron microscopy. HepG2 cells underwent treatment with a range of LDEV concentrations, followed by the evaluation of cytotoxicity and apoptotic gene expression through the MTT assay and semi-quantitative RT-PCR methodologies. A notable cytotoxic effect was recorded at a concentration of 100 μg/mL (P<0.05). Additionally, treatment with 50 and 100 μg/mL LDEVs markedly elevated the apoptotic index (bax/bcl-2 ratio), thereby facilitating the demise of cancer cells. These data exhibited that LDEVs possess the capacity to induce apoptosis in hepatocellular carcinoma cells, indicating their potential utility as functional microbiota-derived anticancer agents and underscoring the significance of BEVs in the domain of cancer therapy (Behzadi et al., 2017). Non-small cell lung cancer (NSCLC) is frequently characterized by its aggressive nature, exhibiting substantial proliferation and metastatic capabilities. The study investigated the involvement of the ferroptosis-associated gene SLC7A11 in NSCLC and assessed engineered BEVs that encapsulate SLC7A11-targeting siRNA as a potential therapeutic intervention. Analysis of The Cancer Genome Atlas (TCGA) and Gene Expression Omnibus (GEO) datasets revealed a significant upregulation of SLC7A11 in NSCLC tissues. The attenuation of SLC7A11 expression in NSCLC cell lines resulted in diminished proliferation, migration, and invasion, while xenograft models corroborated the findings of reduced tumor growth and metastasis. The BEVs were specifically engineered with a lung cell-targeting peptide to facilitate the effective delivery of siSLC7A11, demonstrating both structural integrity and biosafety alongside targeted delivery capabilities. Treatment involving BEVs-LCTP-siSLC7A11 significantly inhibited tumor progression and activated ferroptosis pathways, as evidenced by the altered expression levels of SLC7A11 and transferrin. These observations indicated that engineered BEVs aimed at SLC7A11 present a promising therapeutic strategy for NSCLC (Wan et al., 2025).

The densely populated extracellular matrix (ECM) rich in hyaluronic acid (HA) and the hypoxic nature of the TME pose significant obstacles to effective drug delivery and the infiltration of immune cells. Another research devised an innovative tumor immunotherapy platform utilizing genetically and chemically modified bacterial OMVs that express hyaluronidase (HAase) for the targeted administration of CD73 siRNA (siCD73). The degradation of the ECM facilitated by HAase significantly improved tumor penetration and retention, while the surface modification with 3-aminophenylboronic acid (PBA) allowed for sialic acid-mediated targeting of tumors and enhanced lysosomal escape. The therapeutic intervention resulted in a 4.6-fold inhibition of CD73, thereby impeding the migration, invasion, and adhesion of tumor cells and cancer-associated fibroblasts (CAFs) through the downregulation of EGFR, MMP2/9, and VEGF. The remodeling of the ECM lessened hypoxic conditions, curtailed CAF activation and angiogenesis, and fostered immune responses via the polarization of M1 macrophages, maturation of dendritic cells, and the infiltration of NK and cytotoxic T cells. This platform successfully reprogrammed the immunosuppressive TME, demonstrating substantial anti-tumor efficacy against primary, recurrent, and metastatic tumors (Cheng et al., 2026).

BEVs derived from the *Escherichia coli* strain A5922 were investigated as a potential therapeutic modality against HT-29 colon carcinoma cells. The application of BEVs elicited oxidative stress and instigated mitochondrial autophagy (mitophagy), a process deemed critical for the initiation of cytotoxic effects. The occurrence of mitophagy culminated in the demise of adenocarcinomic cells and the suppression of cellular proliferation. This phenomenon was characterized by an augmented production of reactive oxygen species, a decrement in mitochondrial membrane potential, and an upregulation of PINK1 expression, thereby validating oxidative stress–mediated cytotoxicity. From a mechanistic standpoint, BEVs engaged the Akt/mTOR signaling cascade, effectively linking cellular stress to the induction of apoptosis. Collectively, these data indicated that BEVs possess the capacity to selectively provoke mitophagy and oxidative-stress–mediated cell death in CRC cells, underscoring their promise as a novel therapeutic instrument for the management of CRC and potentially for cancer prophylaxis strategies (Marzoog et al., 2023). *Escherichia coli*-derived OMVs have been identified as nanoscale vehicles for cancer therapeutics; however, traditional methods for cargo loading are frequently intricate. A study introduced a straightforward and scalable technique for the engineering of OMVs that encapsulate overexpressed pre-miRNAs. OMVs generated from the modified E. coli cells incorporated tRNALys-pre-miRNA, which could be directly administered to neoplastic cells. Within eukaryotic cells, the pre-miRNA underwent processing into mature miRNA utilizing a “tRNA scaffold” disguise, thereby facilitating functional gene regulation. *In vivo* experiments demonstrated that targeted OMVs containing pre-miR-126 effectively attenuated the expression of the oncogene CXCR4 and markedly impeded the proliferation of breast cancer cells.

In addition to BEVs, increasing attention has also been directed toward host- and tumor-derived extracellular vesicles, which similarly contribute to intercellular communication and cancer progression within the tumor microenvironment.

Another study meticulously characterized EVs originating from gastric epithelial cells (AGS) subjected to infection by both high- and low-virulence strains of *H. pylori*, employing liquid chromatography-tandem mass spectrometry (LC-MS/MS) methodologies (Subsomwong et al., 2025). The EVs obtained from the infected cellular populations exhibited between 55 and 107 proteins that were differentially expressed in comparison to uninfected control samples. Proteomic and bioinformatic assessments elucidated critical proteins associated with *H. pylori* survival, pathogenicity, metabolic processes, host-cell invasion, and virulence factors. It is particularly noteworthy that EVs derived from AGS cells infected with high-virulence strains were found to be enriched in proteins linked to gastric cancer, such as ATP6V0A1, GAPDH, HINT1, LYZ, and RBX1. These observations contribute to a robust profile of both bacterial and host-derived EV proteins and underscore their potential utility as liquid biopsy biomarkers for the detection of *H. pylori* infection and the assessment of gastric cancer risk, thereby presenting a promising non-invasive strategy for early detection and prognostic evaluation (Subsomwong et al., 2025).

The aim of study was to assess stool-derived EVs as a viable source for the detection of CRC precursors and refined methods for EV isolation. A variety of concentration techniques, including ultrafiltration (10–100 kDa) and speed vacuum, were systematically compared, yielding no statistically significant differences in the recovery of EVs, RNA content, or protein contamination levels. Proteomic analyses revealed a heterogeneous bacterial proteome, whereas host-derived proteins, notably pancreatic enzymes and zymogen granules, were found to be more prevalent. A total of ten surface proteins were identified as promising candidates for the selective isolation of colon-derived EVs. These data provided a foundational framework for the development of immunocapture-based methodologies aimed at enriching stool EVs, thereby enhancing the identification of sensitive EV-based molecular biomarkers for early detection of CRC and screening for precursors in forthcoming research endeavors (Northrop-Albrecht et al., 2025).

Tumor cell-derived EVs possess the capability to modulate immune responses and facilitate metastasis in CRC. This investigation elucidated that CRC-derived EVs (CRC-EVs) activate the cytoplasmic pattern-recognition receptor NOD1 within macrophages, thereby inducing the secretion of inflammatory cytokines and chemokines. Macrophages activated by NOD1 demonstrated an enhanced capacity for CRC cell migration, whereas NOD1-deficient murine models showed a diminished incidence of liver metastasis following CRC-EV treatment. Mechanistically, cell division cycle 42 (CDC42) encapsulated within CRC-EVs is responsible for the activation of NOD1 in macrophages. Moreover, EVs isolated from the plasma of CRC patients exhibiting liver metastasis similarly instigated NOD1 activation in peripheral blood mononuclear cells. Clinically, elevated expression levels of NOD1 in tumor tissues were found to correlate with unfavorable prognostic outcomes. These findings suggested that CRC-EVs facilitate metastasis via NOD1 signaling, thereby proposing NOD1 as a prospective biomarker and therapeutic target for colorectal cancer (CRC) liver metastasis (Wei et al., 2022).

Dysregulated metabolic processes represent a fundamental characteristic of malignancy, and EVs encapsulate metabolomic profiles that are indicative of tumor biology as well as host-microbiome interactions. A study conducted a comprehensive analysis of metabolites in fecal EVs collected from 28 patients diagnosed with solid tumors and 7 healthy control subjects, employing LC-MS–based targeted metabolomics methodologies. Notable discrepancies were identified between the patient cohort and the control group, with L-glutamic acid exhibiting considerable enrichment, while guanine and N-acetylneuraminate displayed significant depletion within tumor-associated EVs. Metabolite Set Enrichment Analysis established connections between these metabolic alterations and pathways encompassing arginine biosynthesis, glyoxylate and dicarboxylate metabolism, along with branched-chain amino acid and unsaturated fatty acid biosynthesis. Receiver Operating Characteristic (ROC) analysis underscored glutamic acid as the most distinguishing biomarker for cancer. A subset of metabolites is likely derived from gut microbiota, thereby underscoring the significance of host-microbiome interactions in this context (Mishra et al., 2025a).

The selective labeling of EVs is of paramount importance for both diagnostic and therapeutic purposes. The aim of one study was to determine two innovative tetranuclear polypyridylruthenium (II) complexes, designated Rubb7-TNL and Rubb7-TL, as phosphorescent stains for EVs. These staining agents demonstrated a marked specificity for EVs in contrast to cellular components, exhibiting robust phosphorescence, enhanced photostability, and negligible leaching until uptake by targeted cells occurred. Utilizing EVs derived from THP-1 monocytic cells, either unstimulated or stimulated with LPS, pivotal observations included: LPS prompted an increase in both the quantity and dimensions of EVs; the EVs maintained their intrinsic physical characteristics post-staining; and the stains were effective in differentiating intact EVs from synthetic liposomes while delineating distinct EV subpopulations based on biochemical variances. Comparable staining patterns observed in EVs derived from A549 epithelial cells indicated a wide-ranging applicability. These results shown that Rubb7-TNL and Rubb7-TL specifically target RNA within EVs, thereby providing a powerful instrument for the characterization and analysis of EVs (Wardhani et al., 2024).

Gene-delivered enzyme prodrug therapy (GDEPT) employs prodrugs that are activated by gene products derived from bacterial or viral sources to achieve selective cytotoxicity against tumors while minimizing adverse effects. Another study progressed the field of GDEPT by utilizing EVs for the delivery of *in vitro* transcribed (IVT) HChrR6 mRNA, specifically targeting HER2-positive breast cancer. The prodrug CB1954 (tretazicar), which has established safe dosage parameters in humans, was transformed into its cytotoxic derivative through the expression of HChrR6 originating from the mRNA delivered by EVs. The verification of functionality was contingent upon the fluorescence produced from the conversion of CNOB, thereby validating the competency of the mRNA. The systemic administration of these IVT mRNA-encapsulated EVs (“IVT EXO-DEPTs”) in conjunction with tretazicar resulted in the growth arrest of HER2-positive xenografts in murine models without eliciting off-target toxicity, thus circumventing the necessity for direct gene injection. This methodology exemplifies a secure, targeted, and clinically applicable strategy for the EV-mediated activation of prodrugs in oncological therapeutics (Forterre et al., 2020).

The studies reviewed in this section provide strong evidence that the molecular cargo of BEVs plays roles in cancer progression, immune modulation, therapeutic response, and biomarker identification. However, there is significant methodological heterogeneity within the current literature, complicating direct comparisons among studies and hindering immediate translational applications. A key difference lies in the biological sources of EVs examined. Some research focused on patient-derived biofluids like urine, stool, serum, or gastric juice to identify microbial EV signatures with clinical relevance (Yoon et al., 2023; Mishra et al., 2025a; You et al., 2025), while others primarily used *in vitro* cell culture systems or murine tumor models to explore mechanistic pathways and therapeutic implications (Behzadi et al., 2017; Cui et al., 2022; Marzoog et al., 2023; Wan et al., 2025; Cheng et al., 2026). Although clinical cohort studies provide valuable translational insights, many are observational and feature limited sample sizes, which impairs statistical power and generalizability. In contrast, mechanistic studies often exhibit stronger experimental control but operate under artificial conditions that may not accurately reflect the complexities of the human tumor microenvironment.

Another significant source of variability is found in the methodologies for EV isolation and characterization. The studies employed varied approaches, including ultracentrifugation, ultrafiltration, precipitation-based methods, and size-exclusion techniques. Several studies did not provide sufficient methodological details concerning EV purity assessments, contaminant exclusion, or adherence to MISEV guidelines, which is crucial, as bacterial EV preparations can contain soluble products, membrane fragments, or endotoxins that might independently affect inflammatory and oncogenic signaling. As a result, some biological effects observed may not solely stem from intact EV cargo. Furthermore, only a limited number of studies utilized rigorous characterization techniques, such as transmission electron microscopy, nanoparticle tracking analysis, proteomics, or RNA profiling, to ensure the integrity and molecular composition of the vesicles, affecting reproducibility and comparability across studies.

Analytical focuses varied significantly among the studies as well. Investigations aimed at identifying clinical biomarkers predominantly employed metagenomic sequencing and diversity analyses to detect microbial signatures associated with cancer (Yoon et al., 2023; You et al., 2025), while others concentrated on proteomics (Subsomwong et al., 2025), metabolomics (Mishra et al., 2025a), or functional cargo delivery systems like miRNAs and siRNAs (Cui et al., 2022; Wan et al., 2025). This diversity in multi-omics approaches, while highlighting the extensive functional landscape of BEVs, also leads to inconsistencies in analytical pipelines, normalization methods, and criteria for interpretation. Notably, many biomarker studies lacked external validation cohorts or longitudinal follow-ups, making it challenging to ascertain whether the identified EV signatures are robust enough for clinical use.

Mechanistic studies exploring immune modulation and tumor progression provided stronger causal inferences due to pathway-specific analyses. For instance, OMVs derived from *Fusobacterium nucleatum* have been linked to resistance to immunotherapy through the activation of the TDO2/AHR pathway in tumor-associated macrophages, and EVs from colorectal cancer (CRC) cells promoted metastasis via NOD1 signaling. Similarly, engineered therapeutic EVs demonstrated promising preclinical efficacy in targeted siRNA delivery, ferroptosis induction, and tumor microenvironment remodeling (Cui et al., 2022; Wan et al., 2025). However, most of these investigations were conducted in murine models or on a limited number of cancer cell lines, so their translational implications remain preliminary. Additionally, dosing strategies, biodistribution, long-term toxicity, and immunogenicity assessments varied across studies.

A crucial methodological challenge is differentiating causation from association in microbiome-EV studies. While several reports have identified disease-specific microbial EV signatures in cancer patients (Yoon et al., 2023; Mishra et al., 2025a; You et al., 2025), it remains unclear whether these alterations contribute directly to tumorigenesis or are simply reflections of cancer-associated dysbiosis. This uncertainty is exacerbated by the dynamic nature of the microbiome, which is influenced by factors such as diet, geography, medication exposure, and host immune status, all of which can affect EV composition independently of cancer biology.

## 4 Diagnostic potential of BEVs

**Figure 3:**
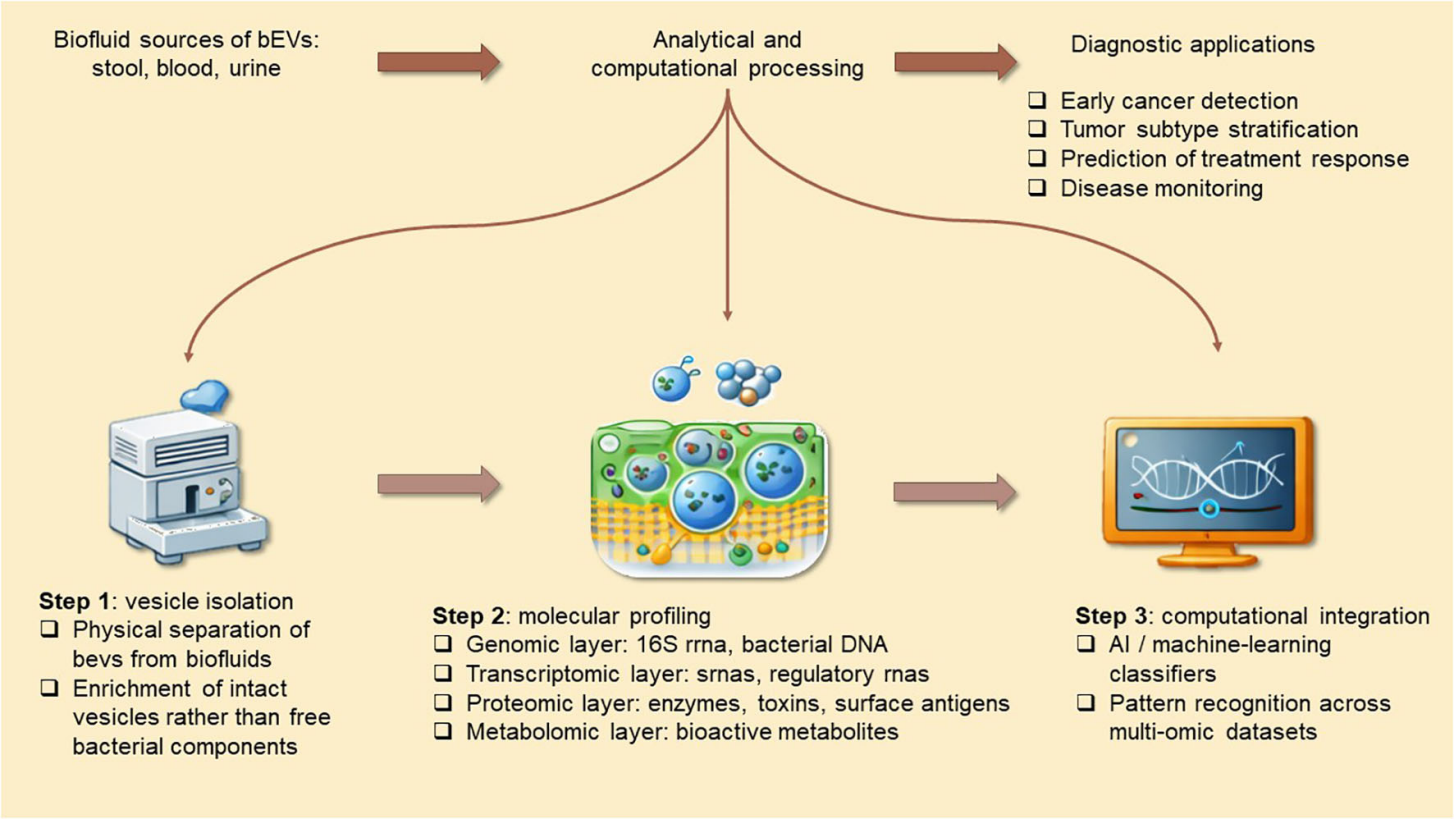
Translational applications of bacterial extracellular vesicles in cancer diagnostics and therapy. This figure illustrates the continuum from non-invasive liquid biopsy to therapeutic engineering of bacterial extracellular vesicles (BEVs). BEVs isolated from biofluids such as stool, blood, and urine can be molecularly profiled to generate diagnostic and prognostic signatures. In parallel, engineered BEVs can be developed as targeted delivery vehicles for immunomodulatory or anticancer agents, highlighting their potential as versatile platforms linking microbiome biology with precision oncology.

BEVs are emerging as promising non-invasive diagnostic biomarkers for cancer. Their presence in biofluids such as feces, blood, and urine, combined with their stable molecular cargo, makes them attractive for liquid biopsy approaches (**Figure 3**).

### 4.1 EV-derived biomarkers in feces, blood, or other biofluids

BEVs can be identified across various biofluids, which reflect both microbial dynamics and host pathophysiological conditions. For instance, fecal EVs exhibit variations in abundance and molecular composition between individuals diagnosed with cancer and those who are healthy, thereby offering prospective biomarkers for the screening of CRC (Zhang et al., 2023). In a similar vein, circulating BEVs present in the bloodstream are correlated with microbial diversity and pathological states; Ou et al. (2023) observed heightened levels of EVs in patients suffering from CRC and inflammatory bowel disease, thereby underscoring their potential as systemic biomarkers. Moreover, BEVs derived from urine have been scrutinized, revealing distinctive microbial compositions in patients with gastric cancer when compared to control subjects, thereby emphasizing the significance of EV profiling in urine for non-invasive cancer detection (Park et al., 2021). Collectively, these investigations imply that BEVs originating from feces, blood, and urine encapsulate critical information regarding disease status and may function as minimally invasive diagnostic biomarkers.

### 4.2 Advanced analytical techniques

The precise detection and comprehensive characterization of BEVs depend on the utilization of sophisticated analytical methodologies. Liquid chromatography coupled with mass spectrometry (LC-MS) permits the sensitive profiling of EV proteins and metabolites, facilitating the identification of cancer-associated biomarkers with considerable specificity (Su et al., 2022). Flow cytometry, particularly in its nano-flow variant, enables the quantification and phenotypic characterization of distinct EV subpopulations, effectively differentiating BEVs from those of the host based on surface markers such as LPS (Mishra et al., 2025a). Sequencing methodologies, which encompass 16S rRNA gene sequencing and metagenomic analysis, provide the capability to profile EV-associated DNA and RNA, thereby identifying taxon-specific signatures and microbial shifts correlated with cancer. Collectively, these technological platforms enhance the isolation, characterization, and quantification of BEVs within clinical specimens, thereby bolstering their potential application in diagnostic endeavors.

Advanced analytical techniques play a critical role in the isolation, quantification, characterization, and validation of BEVs, particularly in studies investigating their diagnostic and therapeutic applications in cancer. Commonly employed approaches include nanoparticle tracking analysis (NTA), dynamic light scattering (DLS), transmission electron microscopy (TEM), scanning electron microscopy (SEM), nano-flow cytometry, proteomics, metagenomic sequencing, metabolomics, and RNA profiling (Kowkabany and Bao, 2024). These methods collectively provide information regarding vesicle morphology, size distribution, concentration, membrane composition, molecular cargo, and bacterial origin. However, despite substantial technological advances, reliable discrimination of bEVs from host- or tumor-derived EVs remains a major methodological challenge, especially in complex biological matrices such as plasma, serum, stool, urine, saliva, and tumor-associated fluids.

One of the major limitations is that bacterial and mammalian EVs frequently overlap in size, density, and biophysical properties. Consequently, conventional size-based isolation techniques such as ultracentrifugation, ultrafiltration, precipitation-based methods, and size-exclusion chromatography are often insufficient for definitive discrimination. In addition, biological samples frequently contain protein aggregates, lipoproteins, apoptotic bodies, microbial debris, and soluble bacterial products that may co-isolate with EV populations and interfere with downstream analyses (Choi and Lee, 2025). This issue is particularly relevant in plasma and serum samples, which contain abundant host-derived EVs and circulating proteins, as well as stool-derived samples that contain dense microbial populations and complex particulate material. Therefore, vesicle purity and specificity may vary substantially depending on both the biological source and the isolation strategy employed (Choi and Lee, 2025).

To improve characterization accuracy, many studies incorporate bacterial-specific molecular signatures to distinguish BEVs from mammalian EV populations. Commonly used bacterial-associated markers include LPS, peptidoglycan, lipoteichoic acid, and outer membrane proteins such as OmpA, OmpC, and porins, which are absent in mammalian EVs (De Langhe et al., 2024). In contrast, host-derived EVs are typically characterized using markers including CD9, CD63, CD81, TSG101, and Alix. In addition, bacterial nucleic acid profiling has emerged as an important complementary strategy for bEV identification. Detection of 16S rRNA, bacterial DNA fragments, microbial small RNAs, and metagenomic signatures can provide further evidence regarding bacterial origin and microbial composition. Several recent studies have also utilized integrated multi-omics approaches combining metagenomics, proteomics, transcriptomics, and metabolomics to improve discrimination specificity and functional characterization.

Advanced analytical platforms such as nano-flow cytometry, high-resolution imaging, immunoaffinity capture systems, and Raman spectroscopy are increasingly being explored to improve sensitivity and reduce contamination from host-derived EV populations. Immunocapture strategies using antibodies targeting bacterial membrane components may further enhance selective enrichment of BEVs from heterogeneous clinical samples. Nevertheless, despite these advances, no universally accepted markers or standardized analytical workflows currently exist for BEV characterization. As a result, substantial variability remains across studies regarding isolation protocols, purity assessment, marker selection, and data interpretation.

### 4.3 Overview of diagnostic potential of BEVs

BEVs represent nanoscale lipid bilayer formations exhibiting inherent anti-tumor characteristics; however, their therapeutic efficacy may be constrained. This investigation employed synthetic bacterial vesicles (SyBV) synthesized from the membranes of Escherichia coli and incorporated a STING agonist (SyBV^STING) to augment immunostimulatory responses. The SyBV^STING instigated dendritic cell activation, thereby fostering substantial Interferon-β secretion, and *in vivo*, it collaboratively inhibited the proliferation of melanoma and colon cancer through the enhancement of tumor-infiltrating T cell populations. Both intratumoral and subcutaneous administrations demonstrated sustained tumor localization exceeding 24 hours with negligible lymphatic dissemination. Toxicological assessments indicated the absence of any histopathological abnormalities (Lötvall et al., 2025).

EVs secreted by oral bacteria exhibit potential utility in the realm of cancer therapeutics; however, their implications for tumor progression remain inadequately elucidated. An investigation developed a larval zebrafish model to assess the effects of EVs derived from *Aggregatibacter actinomycetemcomitans* including both wild-type and mutants deficient in CDT or lipopolysaccharide O-antigen, and *Porphyromonas gingivalis* on the proliferation and metastasis of oral cancer. Tumor cells that were pre-treated with EVs deficient in CDT demonstrated an enhancement in tumor growth, conversely, EVs devoid of LPS O-antigen resulted in a decrease in metastatic spread. EVs originating from P. gingivalis exhibited negligible effects on tumor dynamics. Moreover, EVs from mutant strains of *A. actinomycetemcomitans* also influenced the preferential sites of metastasis. The zebrafish model proved to be both cost-effective and efficient for the *in vivo* investigation of BEVs (Metsäniitty et al., 2024).

CDT, a heterotrimeric virulence factor synthesized by Gram-negative pathogens such as *Campylobacter jejuni*, instigates cell cycle arrest and subsequent cellular demise through its enzymatic component CdtB. EVs facilitate the transmission of CDT from infected cells to those that are uninfected. In another investigation, CDT was observed to target the endo-lysosomal compartment of Caco-2 cells, partially circumventing degradation processes and capitalizing on EV secretion mechanisms. CDT-encapsulated EVs imparted toxin-like effects to both heterologous U937 cells and homologous Caco-2 cells, thereby illustrating successful delivery to intestinal and myeloid tumor cell lines. EVs functioned as principal vectors of active CDT, eliciting moderate apoptotic and autophagic responses, which indicates a delicate equilibrium between cytotoxicity and cellular homeostasis. These observations indicated EV-mediated transfer of CDT as a plausible bacterial-derived biotherapeutic, presenting an innovative approach to CRC management within multidrug therapeutic regimens (Montanari et al., 2022).

In addition to BEVs, increasing attention has also been directed toward host- and tumor-derived extracellular vesicles, which similarly contribute to intercellular communication and cancer progression within the tumor microenvironment.

CRC is significantly modulated by environmental determinants, with alterations in the gut microbiome induced by dietary factors being implicated in the pathogenesis of the disease. This investigation analyzed the metagenomic and metabolomic profiles of stool-derived EVs obtained from 32 CRC patients and 40 healthy control subjects. The application of 16S rDNA sequencing revealed notable alterations in bacterial phyla, particularly within Firmicutes and Proteobacteria, among CRC patients. Metabolomic analysis discerned modifications in seven amino acids, four carboxylic acids, and four fatty acids, encompassing a range from short- to long-chain species. These metabolic alterations were found to correlate with microbial dysbiosis and indicated disrupted amino acid metabolism as a potential mechanistic connection to the development of CRC. Binary logistic regression analysis substantiated the prospective diagnostic significance of these integrated microbial-metabolite signatures. Collectively, EVs derived from the gut microbiome present a promising avenue for the identification of non-invasive biomarkers and elucidation of metabolic contributions to colorectal carcinogenesis (Kim et al., 2020) (**Table 3**).

**Table 3:** Overview of diagnostic potential of bacterial EVs.

Cancer type/model	EV source	Key methods	Key findings/mechanisms	Ref
Colorectal cancer (CRC)	Stool microbial EVs	Metagenomic profiling (16S rDNA sequencing), metabolomics (GC-TOF-MS), binary logistic regression	Altered bacterial phyla (Firmicutes, Proteobacteria), dysregulated amino acids, carboxylic acids, fatty acids; diagnostic model suggests gut dysbiosis is associated with CRC pathogenesis	(Kim et al., 2020)
Melanoma, colon cancer	Synthetic bacterial vesicles (SyBV) from E. coli	EV generation via lysozyme/ionic stress, STING agonist loading, dendritic cell activation, *in vivo* tumour models	SyBVSTING enhances dendritic cell activation and IFN-β production; increases T cell infiltration; suppresses tumour growth without toxicity; promising immuno-oncology approach	(Lötvall et al., 2025)
Zebrafish larvae as a model for studying the impact of oral bacterial vesicles on tumor cell growth and metastasis	Oral cancer	EVs from Aggregatibacter actinomycetemcomitans (wild-type and mutants) and Porphyromonas gingivalis	Zebrafish larval xenotransplantation, EV pretreatment, tumor growth and metastasis analysis	(Metsäniitty et al., 2024)
Extracellular Vesicles from *Campylobacter jejuni* CDT-Treated Caco-2 Cells Inhibit Proliferation of Tumour Intestinal Caco-2 Cells and Myeloid U937 Cells	Colorectal cancer (intestinal and myeloid tumor cells)	Caco-2 cell-derived EVs after CDT treatment	EV isolation, cell proliferation assays, apoptosis/autophagy analysis	(Montanari et al., 2022)

CRC, colorectal cancer; EVs, extracellular vesicles; GC, gastric cancer; HNSCC, head and neck squamous cell carcinoma; STING, stimulator of interferon genes; SyBV, synthetic bacterial vesicles; CDT, cytolethal distending toxin; LPS, lipopolysaccharide; OMV, outer membrane vesicles; NSCLC, non-small cell lung cancer; BEVs, bacterial extracellular vesicles; HA, hyaluronic acid; ECM, extracellular matrix; CAFs, cancer-associated fibroblasts; ROC, receiver operating characteristic; GDEPT, gene-delivered enzyme prodrug therapy; IVT, *in vitro* transcribed; TEM, transmission electron microscopy; NTA, nanoparticle tracking analysis; qPCR, quantitative polymerase chain reaction; LC-MS/MS, liquid chromatography–tandem mass spectrometry; DE, differentially expressed; HChrR6, bacterial prodrug-activating enzyme.

Overall, the studies discussed in this section highlight the growing potential of BEVs as non-invasive diagnostic tools for cancer detection and monitoring. However, when examined more critically, the available evidence is still marked by considerable methodological variability, which makes direct comparison between studies difficult and limits immediate clinical applicability. One of the main differences across these studies is the type of biological sample used. While some investigations focused on stool-derived EVs to assess microbiome-associated changes in colorectal cancer (Kim et al., 2020), others analyzed urine, blood, or circulating EVs to identify systemic microbial signatures linked to cancer development (Ou et al., 2023; Yoon et al., 2023). Although this diversity demonstrates the broad diagnostic potential of BEVs, it also introduces variability in EV composition, microbial content, and analytical sensitivity depending on the source of the sample.

Many of the diagnostic studies relied primarily on metagenomic sequencing and microbial diversity analyses to distinguish cancer patients from healthy controls. For example, the colorectal cancer diagnostic model integrating metagenomic and metabolomic data from stool-derived EVs demonstrated encouraging diagnostic performance (Kim et al., 2020). Nevertheless, the study was conducted in a relatively limited cohort and used a cross-sectional design, which restricts conclusions regarding long-term reproducibility and broader population applicability. Similar limitations were observed in urine-based microbiome EV studies for colorectal cancer, where significant microbial alterations were identified, but external validation and longitudinal analyses were lacking (Yoon et al., 2023). As a result, it remains uncertain whether these microbial EV signatures are stable and reliable enough for routine clinical use.

Another important issue is the lack of consistency in EV isolation and characterization methods. Different studies employed ultracentrifugation, filtration, precipitation-based methods, or density-based separation techniques, often without standardized validation procedures. Since biological fluids contain complex mixtures of host-derived vesicles, protein aggregates, lipoproteins, and bacterial debris, differences in purification strategies can substantially affect downstream analyses and biomarker interpretation. In several studies, EV purity and bacterial specificity were not comprehensively assessed, raising the possibility that some reported findings may partially reflect contaminants rather than true EV-associated cargo. Moreover, only a limited number of investigations thoroughly characterized vesicle morphology, size distribution, or cargo composition using established techniques such as transmission electron microscopy or nanoparticle tracking analysis.

The analytical approaches also varied considerably among studies. Some investigations focused mainly on sequencing-based microbial profiling (Kim et al., 2020; Yoon et al., 2023), whereas others explored advanced nanotechnology-based diagnostic systems, including nanozyme-coated bacterial hybrids and synthetic vesicle platforms designed to enhance sensitivity. While these technologies are innovative and demonstrated promising experimental performance, many were evaluated primarily under controlled laboratory conditions or in preclinical models rather than large clinical populations. Therefore, questions regarding scalability, reproducibility, cost-effectiveness, and real-world clinical implementation remain unresolved.

Several studies also extended beyond diagnostics and investigated the biological functions of bacterial EVs in tumor progression and metastasis. For instance, studies involving toxin-containing EVs or oral bacterial vesicles explored how EV-associated virulence factors may influence tumor growth, immune modulation, or metastatic behavior in zebrafish and cellular models (Montanari et al., 2022; Metsäniitty et al., 2024). Although these studies provided valuable mechanistic insights, their direct relevance as clinically useful diagnostic biomarkers are still preliminary. In addition, experimental systems such as zebrafish larvae or immortalized cell lines cannot fully reproduce the complexity of human tumor biology and host immune responses.

A further limitation across many of these studies is the challenge of distinguishing cancer-specific microbial EV alterations from broader microbiome fluctuations caused by diet, medication use, inflammation, geography, or lifestyle factors. Most cohorts were relatively small and geographically restricted, increasing the risk of bias and reducing external validity. Importantly, only a few studies included independent validation cohorts or longitudinal follow-up analyses, both of which are essential for confirming biomarker stability and clinical reliability.

## 5 BEVs as therapeutic vehicles

BEVs are emerging as versatile therapeutic delivery systems due to their natural ability to transport biomolecules, cross biological barriers, and interact with host cells. Their nanoscale size, biocompatibility, and intrinsic targeting properties make them promising vehicles for delivering therapeutic cargo in cancer and other diseases.

### 5.1 EV-mediated delivery of drugs, miRNAs, prodrugs, or photosensitizers

BEVs possess the capability to encapsulate a diverse array of therapeutic agents, facilitating targeted delivery while concurrently minimizing systemic toxicity. Small-molecule pharmacological agents, including chemotherapeutic compounds, have been incorporated into EVs to augment tumor-specific accumulation and enhance therapeutic efficacy (Hosseini-Giv et al., 2022). In a parallel manner, miRNAs and siRNAs can be effectively delivered via EVs to modulate oncogenic signaling pathways, thereby presenting viable gene therapy strategies for neoplasms that exhibit resistance to traditional treatment modalities (Koeppen et al., 2016). Prodrugs can also be integrated within BEVs to enable controlled release within the TME, leveraging EV-mediated uptake alongside bacterial enzymatic activity for site-specific activation (Liu et al., 2022). Furthermore, BEVs have been investigated for the conveyance of photosensitizers in the context of photodynamic therapy, thereby enhancing cellular uptake and localizing therapeutic effects to tumor tissues while minimizing collateral damage to adjacent healthy cells (Jiang et al., 2023). The inherent characteristics of BEVs, encompassing membrane-bound ligands and surface antigens, facilitate selective engagement with particular cellular phenotypes, thereby augmenting the accuracy of therapeutic interventions. Moreover, EVs can be meticulously engineered to express targeted moieties or to encapsulate combinatorial therapeutic agents, thereby broadening their functional applicability as drug delivery mechanisms. Collectively, these attributes establish BEVs as a highly promising, next-generation therapeutic platform for the treatment of cancer and various other diseases.

### 5.2 Preclinical and *in vivo* studies demonstrating efficacy

Bacterial OMVs are increasingly recognized as promising antitumor agents; however, their clinical application is constrained by issues related to toxicity and the mechanisms underlying their action remain ambiguous. In a study, OMVs were meticulously engineered with cadherin 17 (CDH17) tumor-targeting nanobodies, thereby augmenting tumor specificity while concurrently mitigating adverse effects. These modified OMVs functioned as intrinsic STING (stimulator of interferon genes) agonists, effectively triggering the cGAS-STING pathway in both cancer cells and TAMs. The incorporation of photoimmunotherapy photosensitizers further intensified tumor inhibition and STING activation within TAMs. The synergistic application of nanobody-engineered OMV-mediated photoimmunotherapy in conjunction with CD47 blockade significantly curtailed both primary and metastatic tumors, while also establishing a durable antitumor immune memory. This study elucidates the potential of engineered OMVs as immunotherapeutic STING agonists, presenting a novel strategy to leverage innate immunity for tumor treatment and laying the groundwork for innovative OMV-based cancer immunotherapies (Xia et al., 2025) (**Table 4**).

**Table 4:** Overview of bacterial EVs as therapeutic vehicles.

Key focus	Model/methods	Findings	Ref
Engineered OMVs as STING agonists for tumor immunotherapy	Engineered OMVs with CDH17 nanobodies, loaded with photosensitizers; tested in cancer cells and TAMs	Activated cGAS-STING pathway in cancer cells and TAMs; enhanced tumour inhibition; combined with CD47 blockade suppressed primary and metastatic tumours; established antitumor immune memory	(Xia et al., 2025)
EVs as delivery vehicle for violacein in melanoma therapy	Isolated EVs from *J. lividum*; characterized size, morphology, and protein content; tested on human keratinocytes and melanoma cell lines	EVs efficiently delivered violacein to cancer cells; cargo retained bioactivity; environmentally friendly production	(Kowalska et al., 2024)
Large-scale production of E. coli OMVs for immunotherapy	Isolated OMVs from 160 L culture via metal precipitation and size-exclusion chromatography; tested in mouse syngeneic tumour models	OMVs activated cancer antigen-specific CD8+ T cells; induced tumour remission; synergized with anti-PD-1 therapy	(Won et al., 2023)
Non-toxic artificial OMVs (SyBV) for cancer vaccine	Produced SyBV via lysozyme and high pH treatment; combined with tumour EVs; tested in melanoma-bearing mice	SyBV elicited strong Th1 T cell response; tumour regression; synergistic effect with anti-PD-1	(Park et al., 2021)
Reprogramming tumor microenvironment (TME) using biomineralized bacterial outer membrane vesicles (OMVs) for anticancer therapy	OMVs coated with calcium phosphate (CaP) shells; intravenous administration; evaluation of macrophage polarization (M2-to-M1) and TME modulation; integration with functional components like folic acid or photosensitizers	CaP-coated OMVs safely reprogrammed the immunosuppressive TME, neutralized acidic conditions, promoted M2-to-M1 macrophage polarization, and enabled synergistic combination therapies without systemic toxicity	(Qing et al., 2020)

OMVs, bacterial outer membrane vesicles; TME, tumor microenvironment; CaP, calcium phosphate; STING, stimulator of interferon genes; cGAS, cyclic GMP-AMP synthase; TAMs, tumor-associated macrophages; BGs, bacterial ghosts; BEVs, bacterial extracellular vesicles; SyBV, synthetic bacterial vesicles; EVs, extracellular vesicles; LC-MS/MS, liquid chromatography-tandem mass spectrometry; PD-1, programmed death-1.

Violacein, a naturally occurring pigment derived from indole that exhibits anticancer activity, demonstrates limited solubility in aqueous media, often necessitating the extraction via organic solvents. Another research explored the potential of EVs derived from *Janthinobacterium lividum* as effective carriers for violacein, thereby facilitating stable delivery in aqueous environments. The EVs encapsulating violacein exhibited an average size of 124 nm and were found to contain 932 proteins, a significant proportion of which are of intracellular origin, indicating a biogenesis linked to cellular lysis. Functional assays conducted with human keratinocytes and melanoma cells validated that violacein delivered via EVs interacts effectively with mammalian cells while preserving its anticancer properties. The production of violacein utilizing EVs proved to be more efficient and environmentally sustainable compared to traditional intracellular extraction methods (Kowalska et al., 2024).

The large-scale production of BEVs presents a significant obstacle in their advancement as cancer immunotherapeutic strategies. An investigation delineated a scalable approach for the generation of *Escherichia coli* OMVs through the utilization of metal precipitation and size-exclusion chromatography, resulting in an output of 357 mg of OMVs (~3.93×10^15^ particles) derived from a culture volume of 160 liters. In murine syngeneic tumor models, these OMVs were observed to elicit complete tumor remission and promote the infiltration of CD8^+^ T cells, with a particular emphasis on cancer antigen-specific, stem-like CD8^+^ T cells that express TCF-1 and PD-1. The combination of OMVs with anti-PD-1 therapy exhibited substantial potential as innovative agents in cancer immunotherapy (Won et al., 2023). Synthetic bacterial vesicles (SyBV) have been engineered as a low-toxicity substitute for natural OMVs in the context of cancer immunotherapy. Generated through the application of lysozyme and elevated pH conditions, SyBV are devoid of RNA, DNA, and cytosolic impurities, thereby circumventing systemic pro-inflammatory reactions in murine models while preserving the capacity for dendritic cell activation. The co-immunization strategy employing SyBV alongside tumor-derived EVs instigated melanoma regression via Th1-mediated T cell responses and a well-regulated production of antibodies. Furthermore, the administration of anti-PD-1 therapy significantly potentiated these immunological effects. SyBV exhibited superior adjuvant efficacy compared to traditional adjuvants, thereby presenting a secure and efficacious platform for the induction of tumor-specific immune responses (Park et al., 2021). Bacterial OMVs were meticulously modified by incorporating calcium phosphate (CaP) shells to effectively reprogram the immunosuppressive TME. The CaP coating serves to inhibit antibody-mediated clearance and mitigate OMV toxicity, neutralize the acidic characteristics of the TME, and facilitate the polarization of macrophages from the M2 to the M1 phenotype, thereby augmenting antitumor immune responses. The functionalization of these vesicles with folic acid or photosensitizers paves the way for combination therapies, thereby illustrating a versatile and efficacious OMV-based platform for synergistic cancer treatment (Qing et al., 2020).

Overall, the studies summarized in this section demonstrate the considerable therapeutic potential of BEVs in cancer treatment, particularly as immunomodulatory agents and targeted delivery platforms. However, despite the promising preclinical findings, the available evidence remains highly heterogeneous from a methodological standpoint, and several limitations currently restrict translation into clinical practice. Most importantly, nearly all studies were conducted *in vitro* or in animal models, meaning that the observed therapeutic efficacy has not yet been validated in human clinical settings.

One major strength across these studies is the diversity of engineering strategies used to enhance the antitumor properties of BEVs. Several investigations focused on modifying bacterial OMVs to improve tumor targeting, reduce toxicity, or stimulate immune activation. For example, engineered OMVs expressing tumor-targeting nanobodies or carrying STING agonists showed enhanced immune-mediated tumor suppression and improved responses to checkpoint inhibitors in murine models. Similarly, synthetic bacterial vesicles and calcium phosphate-coated OMVs were designed to reduce systemic inflammation while maintaining immunostimulatory activity. These approaches illustrate the flexibility of BEVs as customizable therapeutic systems. However, the methodologies used for vesicle engineering varied substantially between studies, making it difficult to directly compare efficacy, safety, or reproducibility across different platforms.

Another important methodological issue involves the reliance on animal tumor models, particularly murine xenograft and syngeneic models. Although these systems are valuable for evaluating therapeutic responses and immune activation, they do not fully replicate the complexity of human tumor biology, microbiome diversity, or immune interactions. In many studies, treatment responses were evaluated under relatively controlled experimental conditions using homogeneous tumor cell populations, which may overestimate therapeutic efficacy compared to the highly heterogeneous clinical environment observed in human cancers. Furthermore, differences in microbiome composition between animal models and humans may significantly influence the biological behavior of bacterial vesicles and their immunological effects.

The characterization and purification of therapeutic EVs also varied considerably across studies. Different investigations used distinct isolation methods, including ultracentrifugation, filtration, precipitation, or synthetic vesicle generation approaches, often with limited discussion regarding purity, endotoxin contamination, or batch consistency. Since bacterial vesicles naturally contain immunogenic molecules such as LPS and bacterial proteins, incomplete purification may contribute to inflammatory or toxic effects independent of the intended therapeutic cargo. While several studies attempted to reduce toxicity through genetic or chemical engineering, long-term biosafety assessments were generally limited, and systematic evaluations of immunogenicity, biodistribution, and off-target effects were not consistently performed.

The therapeutic cargo delivered by BEVs also differed markedly among studies. Some investigations focused on immune stimulation using STING agonists or immunotherapeutic combinations, whereas others explored RNA-based therapies, including pre-miRNAs, siRNAs, or prodrug-activating mRNAs. Additional studies investigated naturally bioactive bacterial products such as violacein or apoptosis-inducing vesicles derived from *Lactobacillus* species. Although these findings collectively demonstrate the broad therapeutic versatility of BEVs, many studies primarily emphasized proof-of-concept efficacy rather than clinically translatable delivery optimization. Important pharmacological parameters such as dosage standardization, circulation half-life, repeated administration safety, and large-scale manufacturing feasibility were often insufficiently addressed.

Another limitation is that many studies reported strong antitumor effects without extensive mechanistic validation. While several investigations identified immune-related pathways such as cGAS-STING activation, macrophage polarization, or ferroptosis induction, the downstream molecular interactions and long-term consequences of manipulating these pathways remain incompletely understood. In addition, combination therapies involving checkpoint inhibitors or photodynamic therapy frequently demonstrated synergistic effects, but the precise contribution of BEVs relative to the accompanying therapeutic modalities was not always clearly distinguishable.

Scalability and translational feasibility also remain important concerns. Although some studies proposed large-scale OMV production strategies or synthetic vesicle systems to improve clinical applicability, standardized manufacturing protocols and regulatory frameworks for therapeutic bacterial vesicles are still lacking. Batch-to-batch variability, storage stability, and quality control remain major unresolved challenges that could significantly affect future clinical development.

## 6 Host-microbiome interactions and tumor modulation

The interaction between the host and the microbiome plays a critical role in cancer initiation, progression, and therapeutic response. BEVs represent a key communication interface through which the microbiome influences host immunity and tumor biology by reshaping the TME.

### 6.1 Immune modulation and TME reprogramming

**Figure 4:**
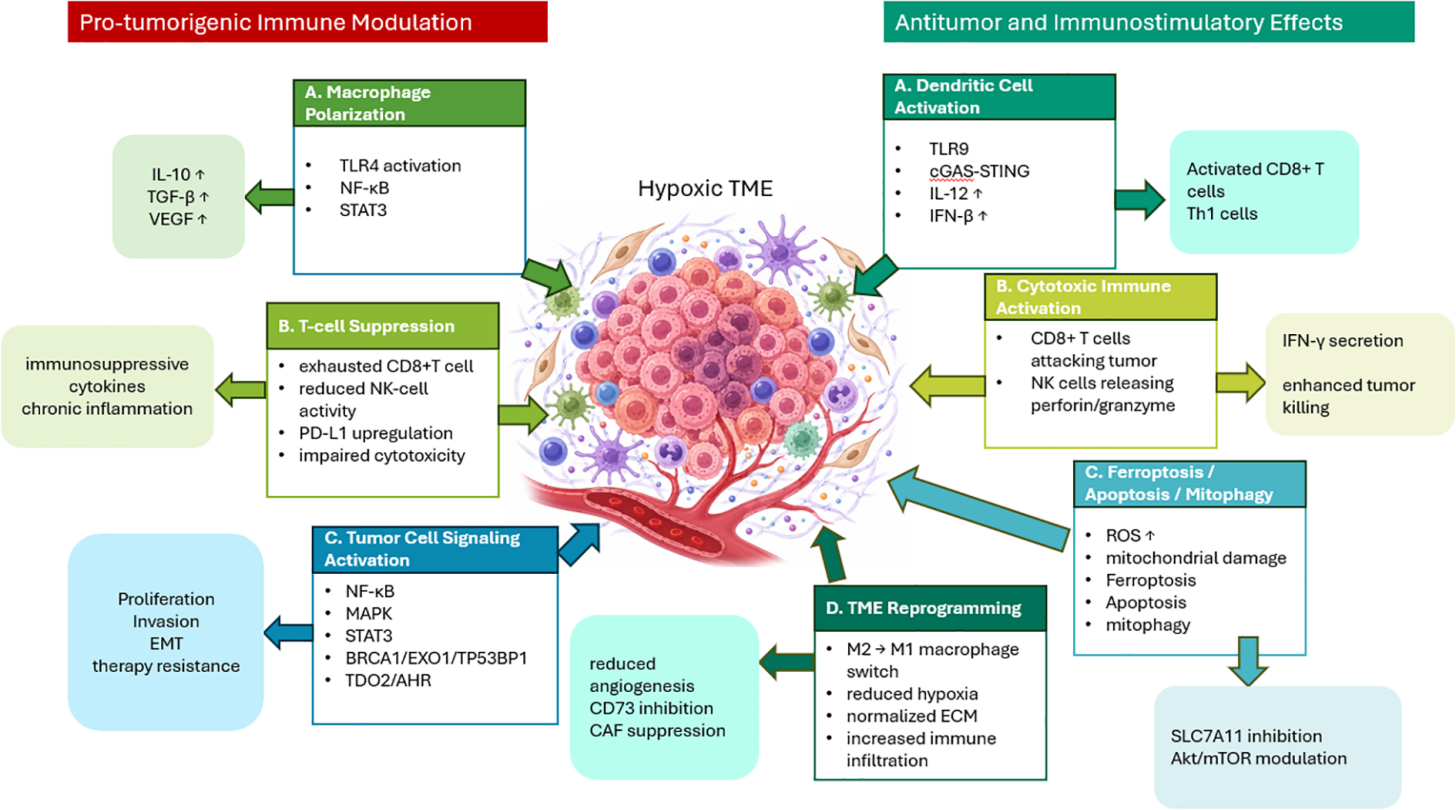
Immune modulation and tumor microenvironment reprogramming mediated by bacterial extracellular vesicles (BEVs). Gut microbiota-derived BEVs carry diverse bioactive cargo, including lipopolysaccharides (LPS), bacterial DNA, small RNAs, metabolites, and toxins, which modulate tumor progression and host immunity. In the tumor-promoting setting, BEVs activate inflammatory and immunosuppressive pathways such as NF-κB, STAT3, and TDO2/AHR, leading to macrophage polarization toward the M2 phenotype, T-cell dysfunction, angiogenesis, extracellular matrix remodeling, and metastasis. Conversely, specific commensal or engineered BEVs stimulate antitumor immunity through activation of TLR9 and cGAS-STING signaling, promoting dendritic cell maturation, Th1/CD8+ T-cell activation, IFN-γ production, ferroptosis, apoptosis, and tumor microenvironment normalization. These findings highlight the dual and context-dependent role of BEVs in cancer progression and therapeutic modulation.

BEVs play a significant role in the modulation of immune responses by transporting bioactive molecules that affect both innate and adaptive immune mechanisms within the TME. These vesicles are equipped with pathogen-associated molecular patterns (PAMPs), such as LPS, lipoteichoic acids, peptidoglycans, and bacterial nucleic acids, which have the capacity to activate pattern recognition receptors (PRRs) including Toll-like receptors (TLRs) and NOD-like receptors located on immune and tumor cells (Kaparakis-Liaskos and Ferrero, 2015). The activation of these signaling pathways may result in chronic inflammation, immune tolerance, or immune suppression, all of which can facilitate the progression of tumors. BEVs have been demonstrated to modulate the polarization of macrophages, thereby facilitating a transition toward an immunosuppressive M2 phenotype that is conducive to tumor proliferation, angiogenesis, and metastasis (Jiang et al., 2022). Moreover, EV-mediated signaling plays a pivotal role in the maturation of dendritic cells and the presentation of antigens, subsequently influencing T-cell activation and orchestrating antitumor immune responses (Kuehn and Kesty, 2005). Aberrant exposure to BEVs has the potential to compromise the functionality of cytotoxic T lymphocytes and natural killer (NK) cells, thereby promoting immune evasion by neoplastic cells (Zitvogel et al., 2018). Within the TME, BEVs also exert regulatory effects on cytokine and chemokine networks. The cargo associated with EVs has been documented to augment the secretion of immunosuppressive cytokines such as IL-10 and TGF-β, while concurrently diminishing pro-inflammatory and antitumor mediators, thus contributing to the reprogramming of the TME (Pickup et al., 2013). Furthermore, BEVs have the capacity to activate various signaling cascades, including NF-κB, STAT3, and STING, which collectively modulate immune cell recruitment, enhance tumor cell viability, and regulate the inflammatory equilibrium (Barber, 2014). Recent research indicates that BEVs can significantly modify the metabolic milieu within the TME by transferring microbial metabolites that influence the metabolism and functionality of immune cells. This metabolic reprogramming further undermines effective antitumor immunity and facilitates tumor advancement (Zhang and Davies, 2016). Collectively, these observations underscore the role of BEVs as pivotal mediators of host–microbiome interactions that reconfigure the TME toward a state conducive to tumor promotion (**Figure 4**).

### 6.2 Crosstalk between gut microbiota, EVs, and systemic cancer pathways

**Figure 5:**
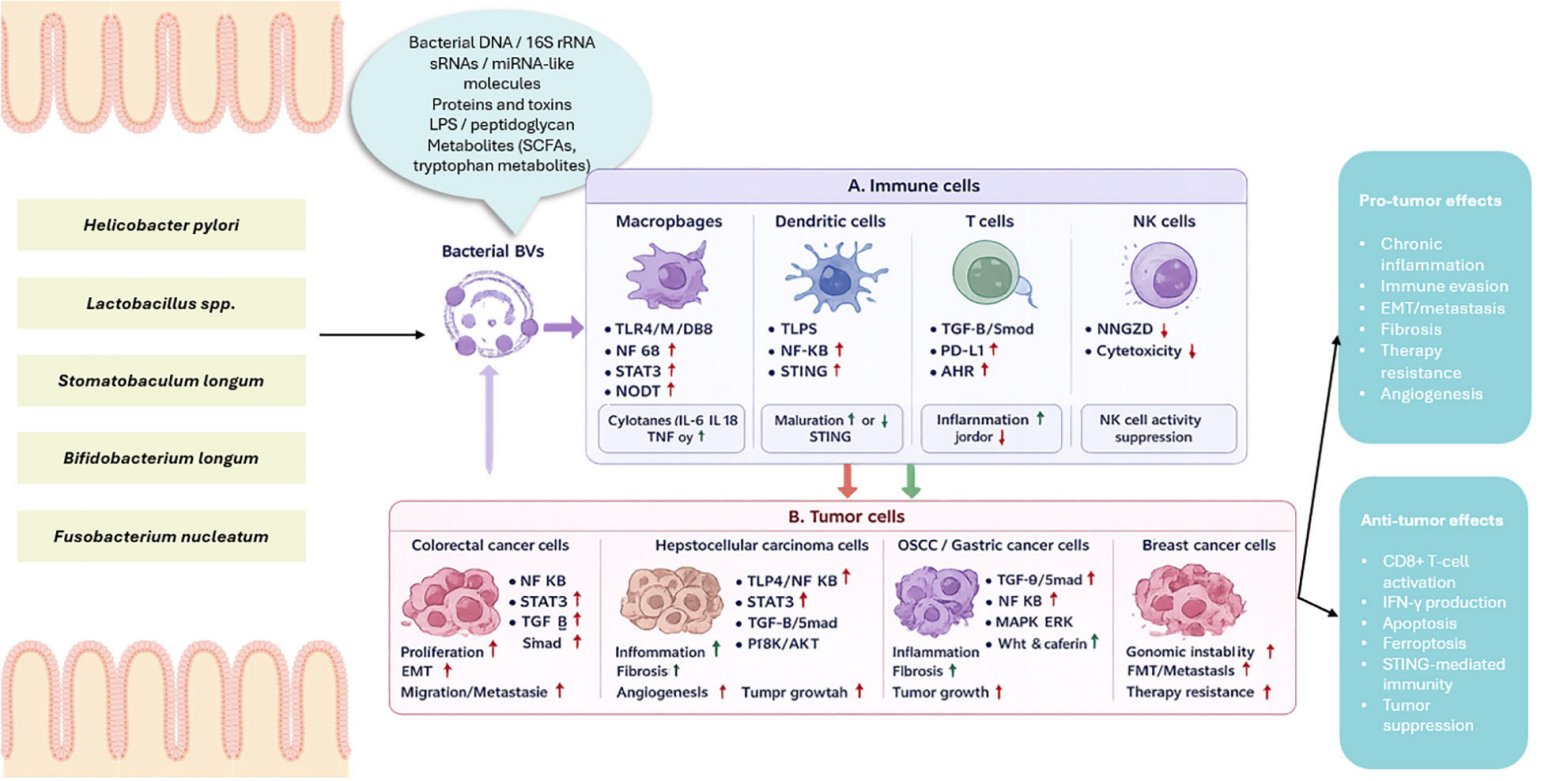
Crosstalk between gut microbiota-derived extracellular vesicles (BEVs) and systemic cancer signaling pathways. Representative gut microbiota, including *Helicobacter pylori, Lactobacillus* spp.*, Stomatobaculum longum, Bifidobacterium longum, and Fusobacterium nucleatum*, release bacterial extracellular vesicles (BEVs) containing diverse bioactive cargo such as bacterial DNA/16S rRNA, sRNAs/miRNA-like molecules, proteins, toxins, lipopolysaccharides (LPS), peptidoglycan, and microbial metabolites. These vesicles interact with immune and tumor cells and modulate multiple signaling pathways involved in inflammation, immune regulation, and tumor progression. In immune cells, BEVs regulate pathways including TLR4/MyD88, NF-κB, STAT3, STING, PD-L1, AHR, and TGF-β/Smad signaling in macrophages, dendritic cells, T cells, and NK cells. In tumor cells, BEVs influence oncogenic pathways associated with colorectal, hepatocellular, gastric/oral squamous, and breast cancers, promoting proliferation, epithelial–mesenchymal transition (EMT), fibrosis, angiogenesis, metastasis, genomic instability, and therapy resistance. Depending on their molecular cargo and cellular targets, BEVs may exert both pro-tumor and anti-tumor effects, including immune suppression or activation of antitumor immunity through STING-mediated responses, IFN-γ production, CD8+ T-cell activation, apoptosis, and ferroptosis.

Oral squamous cell carcinoma (OSCC) exhibits a high prevalence among Asian demographics, with dysbiosis of the oral microbiome potentially playing a significant role in its etiology. An investigation aimed to elucidate the involvement of *Stomatobaculum longum* (*S. longum*) in the progression of OSCC and the underlying mechanisms mediated by BEVs. Analysis of tumor and adjacent non-tumor tissues obtained from OSCC patients through 16S rRNA sequencing revealed a substantial enrichment of *S. longum* within tumor tissues. Both *in vitro* and *in vivo* experimental approaches indicated that *S. longum* facilitated OSCC proliferation and cell cycle advancement, effects that were effectively counteracted by GW4869, a known inhibitor of EV release. EVs derived from S. longum (SBL-EVs) were isolated, characterized, and shown to promote OSCC progression via the activation of the BRCA1/EXO1/TP53BP1 signaling pathway. These findings indicated the oncogenic capacity of *S. longum* through EVs and propose BEVs as innovative therapeutic targets for the treatment of OSCC (Yan et al., 2025) (**Table 5**, **Figure 5**). The gut microbiota has increasingly been associated with the pathogenesis of hepatocellular carcinoma (HCC), potentially through the mediation of BEVs. Another work aimed to elucidate the role of *Bifidobacterium longum*-derived EVs (*B.longum*-EVs) in the progression of HCC. Employing a diethylnitrosamine (DEN)-induced HCC murine model, it was observed that fecal concentrations of B. longum were significantly diminished. *B.longum*-EVs were successfully isolated, labeled, and demonstrated to be internalized by murine hepatocytes (AML12). *In vitro* experiments revealed that *B.longum*-EVs mitigated TGF-β1-induced fibrosis, apoptosis, and oxidative stress in AML12 cells. *In vivo*, the administration of *B.longum*-EVs resulted in a reduction of DEN-induced liver fibrosis, apoptosis, oxidative damage, tumorigenesis, and liver injury, which was accompanied by a downregulation of TGF-β1 expression and Smad3 phosphorylation. These data shown that *B.longum*-EVs confer protection to hepatocytes and may represent a promising therapeutic strategy to avert HCC development through the modulation of fibrosis, apoptosis, and oxidative stress (Li et al., 2024).

**Table 5:** Overview of host-microbiome interactions and tumor modulation.

Key focus	Model/methods	Findings	Ref
*S. longum*-Derived EVs Promote OSCC Malignancy via BRCA1/EXO1/TP53BP1	16S rRNA sequencing of tumor vs. non-tumor tissues, *in vitro* OSCC cell proliferation and cell cycle assays, *in vivo* xenograft models, GW4869 EV inhibitor, EV isolation (ultracentrifugation), TEM, NTA, high-throughput sequencing	S. longum enriched in tumor tissues; promotes OSCC proliferation and cell cycle progression; effects reversed by GW4869; SBL-EVs activate BRCA1/EXO1/TP53BP1 pathway, suggesting EV-mediated oncogenic role	(Yan et al., 2025)
*B. longum*-Derived EVs Prevent HCC via TGF-β1/Smad Modulation	DEN-induced HCC mouse model, real-time PCR for B. longum, EV isolation (ultracentrifugation), PKH26 labeling, AML12 hepatocyte assays (fibrosis, apoptosis, ROS), Western blot, ELISA, flow cytometry, *in vivo* liver histology	B. longum reduced in HCC mice; B. longum-EVs enter hepatocytes and attenuate TGF-β1-induced fibrosis, apoptosis, oxidative stress; *in vivo*, EVs reduce tumor formation, improve liver function, downregulate TGF-β1/Smad3	(Li et al., 2024)
*S. aureus*-Derived EVs Enhance Endocrine Therapy in Breast Cancer	Blood microbiome analysis, tamoxifen treatment of breast cancer cells with/without S. aureus EVs	S. aureus more abundant in healthy controls; EVs increase tamoxifen efficacy; potential use of S. aureus EVs as adjuvants for breast cancer therapy	(An et al., 2022)
*Bacillus* Spores Expressing EV-Derived Tetraspanin Protect Against Liver Fluke	Oral vaccine using recombinant *B. subtilis* spores expressing Ov-TSP-2-LEL, hamster model, serum/bile IgG and IgA measurement, challenge infection with O. viverrini metacercariae	Vaccinated hamsters show strong humoral responses; 56% reduction in adult flukes and fecal eggs; serum IgG blocks fluke EV uptake; demonstrates efficacy of oral EV-derived vaccine against carcinogenic liver fluke	(Phumrattanaprapin et al., 2021)

OSCC, oral squamous cell carcinoma; S. longum, Stomatobaculum longum; EVs, extracellular vesicles; SBL-EVs, S. longum-derived extracellular vesicles; HCC, hepatocellular carcinoma; B. longum, Bifidobacterium longum; AML12, murine hepatocyte cell line; ROS, reactive oxygen species; ELISA, enzyme-linked immunosorbent assay; S. aureus, Staphylococcus aureus; Ov-TSP-2, Opisthorchis viverrini tetraspanin-2; LEL, large extracellular loop; IgG, immunoglobulin G; IgA, immunoglobulin A.

The human microbiome, which plays a critical role in the metabolism of estrogen and is referred to as the estrobolome, may have significant implications for breast cancer therapy. The aim of study was to investigate the composition of the blood microbiome in healthy individuals and patients diagnosed with breast cancer, with a particular emphasis on bacteria that produce the enzymes β-glucuronidase and β-galactosidase, both of which are crucial in the process of estrogen metabolism. Notably, Staphylococcus species exhibited a higher prevalence in healthy individuals and were subsequently selected for further analysis. The impact of Staphylococcus aureus on endocrine therapy was scrutinized in conjunction with the administration of tamoxifen. Analysis of the blood microbiome indicated elevated levels of β-glucuronidase-producing bacteria in patients with breast cancer. Significantly, EVs originating from *S. aureus* were found to enhance the anti-cancer efficacy of tamoxifen in experimental models. These results indicated that S. aureus-derived EVs may augment endocrine therapy, underscoring their potential as adjuvants in breast cancer treatment and affirming the estrobolome as a viable target for improving therapeutic outcomes (An et al., 2022). The liver fluke *Opisthorchis viverrini* is responsible for hepatobiliary disorders and elevates the likelihood of bile duct carcinogenesis in Southeast Asia. In an effort to mitigate infection and the resultant pathological conditions, an oral vaccine has been engineered utilizing recombinant Bacillus subtilis spores that express the large extracellular loop (LEL) of *O. viverrini* tetraspanin-2 (Ov-TSP-2), a protein that is prevalent on EVs secreted by the fluke. Prior to challenge infection with metacercariae, hamsters underwent oral immunization with the recombinant spores. The immunized hamsters demonstrated the production of serum IgG and bile IgG/IgA targeting Ov-TSP-2-LEL, and their serum IgG was capable of inhibiting the uptake of fluke EVs by human bile duct epithelial cells. Immunization led to a 56% decrease in both adult fluke populations and fecal egg excretion when compared to control groups. These observations substantiate the efficacy of oral vaccination utilizing Ov-TSP-2-LEL spores, thereby endorsing its potential application as a vaccine for the management of carcinogenic liver fluke infections in humans (Phumrattanaprapin et al., 2021).

## 7 Challenges, limitations, and future perspectives

### 7.1 Technical challenges in EV isolation and standardization

Notwithstanding the increasing scholarly attention directed toward BEVs as potential diagnostic and therapeutic modalities in the realm of oncology, substantial technical obstacles persist. Techniques employed for the isolation of EVs, including ultracentrifugation, size-exclusion chromatography, and immunoaffinity capture, yield vesicles characterized by inconsistent purity, composition, and recovery efficiencies. The absence of standardized protocols significantly undermines reproducibility across various laboratories, thereby complicating the direct comparative analysis of research findings. Furthermore, contamination with bacterial remnants, LPS, or other extracellular entities may obfuscate subsequent molecular investigations. The quantitative characterization of EVs is additionally complicated by the inherent heterogeneity in vesicle size, cargo, and membrane composition, which renders precise functional assays exceedingly challenging. Consequently, the establishment of validated, high-throughput, and reproducible methodologies for the isolation and characterization of EVs is imperative for the advancement of EV-based cancer diagnostics.

A major methodological limitation in the current field is the difficulty in accurately distinguishing BEVs from host- or tumor-derived EVs in complex biological samples such as blood, stool, urine, and tumor-associated fluids. Because these vesicle populations often overlap considerably in size, density, morphology, and biophysical characteristics, commonly used isolation techniques including ultracentrifugation, filtration, and size-exclusion chromatography are frequently insufficient for definitive discrimination. Although several studies have attempted to improve specificity using bacterial-associated markers such as LPS, peptidoglycan, outer membrane proteins, and bacterial nucleic acid signatures including 16S rRNA, no universally accepted or standardized identification strategy currently exists. In addition, contamination with host EVs, protein aggregates, bacterial debris, or soluble microbial products may influence downstream proteomic, metabolomic, and sequencing analyses, thereby complicating interpretation of biological and diagnostic findings. Consequently, the lack of standardized isolation and characterization protocols remains a significant challenge that may affect reproducibility, cross-study comparability, and the translational reliability of BEV-based diagnostic and therapeutic applications.

### 7.2 Translational hurdles for clinical implementation

The transition of BEVs from preclinical investigation to clinical application encounters numerous translational impediments. Concerns regarding safety, particularly with respect to immunogenicity and the potential for systemic inflammation, necessitate meticulous consideration. The regulatory framework governing biological nanomaterials remains inadequately developed, thereby engendering uncertainty surrounding clinical trial execution. Furthermore, the scalability for the mass production of engineered EVs, the preservation of cargo stability, and the assurance of batch-to-batch consistency represent critical challenges. It is imperative to establish robust preclinical models, such as humanized or organoid systems, to rigorously assess efficacy and toxicity prior to their translation into human studies.

### 7.3 Potential for multi-omics integration and personalized cancer therapy

BEVs present unparalleled prospects for precision oncology when they are amalgamated with multi-omics methodologies, encompassing genomics, transcriptomics, proteomics, and metabolomics. Integrated analyses can elucidate patient-specific EV signatures, facilitating early detection, prognostication, and therapeutic monitoring. Furthermore, the engineering of EVs to incorporate therapeutic payloads such as microRNAs, immunomodulators, or prodrugs permits personalized interventions aimed at tumor-specific signaling pathways. Future investigations should prioritize the alignment of multi-omics frameworks with EV diagnostics and therapeutics to optimize translational efficacy and fully harness their potential in tailored cancer management.

## 8 Conclusions

Gut microbiota-derived extracellular vesicles (BEVs) have emerged as important mediators of host–microbiome communication and play a complex role in cancer initiation, progression, immune modulation, and therapeutic response. Through the transfer of bioactive cargo, including bacterial nucleic acids, proteins, toxins, lipopolysaccharides, and microbial metabolites, BEVs regulate multiple signaling pathways involved in tumor biology, such as NF-κB, STAT3, STING/cGAS, TGF-β/Smad, PI3K/AKT, MAPK/ERK, and Wnt/β-catenin signaling. Accumulating evidence indicates that microbiota-derived EVs can exert both pro-tumorigenic and anti-tumorigenic effects depending on their microbial origin, molecular composition, and target cell interactions. Pro-tumorigenic BEVs contribute to chronic inflammation, immune evasion, epithelial–mesenchymal transition (EMT), angiogenesis, fibrosis, metastasis, genomic instability, and therapy resistance. In contrast, beneficial microbiota-derived EVs may activate antitumor immunity through STING-mediated signaling, IFN-γ production, CD8+ T-cell activation, apoptosis, and ferroptosis induction. These findings highlight the dual and context-dependent nature of BEV-mediated cancer regulation. Despite growing interest in the field, several major challenges remain, including limited standardization of EV isolation and characterization methods, difficulties distinguishing bacterial EVs from host-derived EV populations, incomplete mechanistic understanding, and insufficient translational and clinical evidence. Future studies integrating multi-omics technologies, advanced imaging, functional validation, and standardized EV profiling approaches will be essential to better define the biological and clinical significance of microbiota-derived EVs.

This molecular composition empowers BEVs to function as non-invasive and highly informative biomarkers for the purposes of cancer detection, prognostication, and the assessment of therapeutic efficacy. Sophisticated profiling methodologies, such as metabolomics, proteomics, and RNA sequencing, have elucidated distinctive EV signatures across a spectrum of cancers, underscoring their potential as sensitive and specific diagnostic instruments. In addition to their diagnostic applications, BEVs can be customized or utilized as precision delivery systems for anti-cancer agents, immunomodulatory compounds, and RNA-based therapeutics. The capacity of BEVs to modulate the TME, augment immune responses, and selectively deliver therapeutic agents to tumor cells emphasizes their potential in the realm of personalized cancer therapy. The amalgamation of bacterial EV-derived biomarkers with multi-omics platforms further facilitates the formulation of bespoke therapeutic interventions, thereby allowing for more accurate predictions of treatment efficacy and resistance mechanisms. Notwithstanding the prevailing technical and translational obstacles, such as the standardization of EVs and the challenges associated with large-scale production, BEVs signify an innovative frontier in oncology, merging the capabilities of nascent cancer biomarkers with cutting-edge therapeutic modalities. Their bifunctional nature as both diagnostic markers and precision oncology instruments positions BEVs to revolutionize the landscape of cancer management in the foreseeable future.

In summary, accumulating evidence suggests that bEVs play multifaceted roles in cancer biology through their ability to mediate intercellular communication, modulate immune responses, remodel the TME, and transfer biologically active cargo including proteins, nucleic acids, toxins, metabolites, and signaling molecules. Recent studies further highlight the potential of bEVs as minimally invasive diagnostic biomarkers and as promising therapeutic platforms for targeted drug delivery, immune modulation, and combination cancer therapies. Advances in metagenomics, proteomics, nanoengineering, and multi-omics profiling have substantially improved the understanding of bEV-mediated mechanisms and expanded their potential translational applications in oncology. Nevertheless, despite these promising developments, the field remains in an early and methodologically heterogeneous stage. Several important technical and translational limitations continue to restrict reproducibility, cross-study comparability, and clinical implementation. One of the major unresolved challenges is the absence of universally accepted markers for bEV characterization. Unlike mammalian EVs, which are commonly identified using established markers such as CD9, CD63, and CD81, bEV studies frequently rely on bacterial species-specific components including LPS, peptidoglycan, and outer membrane proteins such as OmpA and OmpC. However, these markers are not universally applicable across bacterial species, and no standardized characterization framework currently exists.

Another significant limitation involves the difficulty of distinguishing bEVs from host- or tumor-derived EV populations in complex biological samples. Because bacterial and mammalian EVs overlap considerably in size, density, and biophysical properties, conventional isolation approaches including ultracentrifugation, filtration, and size-exclusion chromatography are often insufficient for definitive discrimination. In addition, contamination with host EVs, lipoproteins, protein aggregates, microbial debris, and soluble bacterial products may influence downstream sequencing, proteomic, and metabolomic analyses. Although advanced analytical approaches such as nano-flow cytometry, immunoaffinity capture, metagenomic sequencing, and integrated multi-omics platforms have improved characterization specificity, standardized analytical workflows and validated bacterial-specific marker panels remain lacking. The complexity of biological matrices also represents an important methodological challenge. Sample sources such as plasma, serum, stool, urine, saliva, and tissue-associated microbiota differ substantially in microbial composition, host EV abundance, protein content, viscosity, and contaminating particles, all of which influence vesicle recovery, purity, and analytical sensitivity. Consequently, no single isolation strategy currently provides optimal performance across all sample types, and methodological variability between studies remains substantial. In addition, many currently available studies are limited by relatively small cohort sizes, insufficient external validation, reliance on preclinical models, and incomplete mechanistic investigation, which collectively reduce the robustness and generalizability of existing findings. Important biosafety concerns also remain insufficiently resolved for therapeutic applications. Because bEVs naturally contain immunogenic bacterial components such as LPS, bacterial toxins, and inflammatory membrane-associated molecules, excessive immune activation, systemic toxicity, and off-target effects remain potential risks. Although several studies have explored engineering strategies including endotoxin reduction, membrane modification, and synthetic or hybrid vesicle design to improve safety profiles, comprehensive long-term biosafety evaluations and standardized regulatory frameworks are still lacking.
